# A model of individualized canonical microcircuits supporting cognitive operations

**DOI:** 10.1371/journal.pone.0188003

**Published:** 2017-12-04

**Authors:** Tim Kunze, Andre D. H. Peterson, Jens Haueisen, Thomas R. Knösche

**Affiliations:** 1 Max Planck Institute for Human Cognitive and Brain Sciences, Leipzig, Germany; 2 Institute of Biomedical Engineering and Informatics, Ilmenau University of Technology, Ilmenau, Germany; 3 Department of Medicine, University of Melbourne, Melbourne, Australia; Plymouth University, UNITED KINGDOM

## Abstract

Major cognitive functions such as language, memory, and decision-making are thought to rely on distributed networks of a large number of basic elements, called canonical microcircuits. In this theoretical study we propose a novel canonical microcircuit model and find that it supports two basic computational operations: a gating mechanism and working memory. By means of bifurcation analysis we systematically investigate the dynamical behavior of the canonical microcircuit with respect to parameters that govern the local network balance, that is, the relationship between excitation and inhibition, and key intrinsic feedback architectures of canonical microcircuits. We relate the local behavior of the canonical microcircuit to cognitive processing and demonstrate how a network of interacting canonical microcircuits enables the establishment of spatiotemporal sequences in the context of syntax parsing during sentence comprehension. This study provides a framework for using individualized canonical microcircuits for the construction of biologically realistic networks supporting cognitive operations.

## Introduction

Most modern neuroscientific theories adopt a connectionist’s approach, where higher cognitive functions are anchored in a distributed network of a large number of similar basic elements, often called *canonical microcircuits* [1–7]. These relatively simple elements give rise to the complexity of cognitive processing by virtue of (i) their interaction in large numbers within an organized network topology, and (ii) the individual tuning of their properties. While higher cognitive operations are necessarily associated with the combination and distribution of large amounts of information and therefore must rely on the connective structure of the wider network, canonical microcircuits may play an important role by providing a set of *basic operations* [2, 8, 9]. In this study, we propose a generic computational framework for a cortical canonical microcircuit. We systematically investigate this model’s ability to represent important basic operations, quantify the influence of fundamental structural features and physiological variables, and demonstrate its capacity to cooperate in larger networks to implement cognitive function.

Two of the most fundamental basic operations at the local level are *signal flow gating* and *working memory*. Signal flow gating controls the transmissibility of neural signals. It is likely to depend (i) in a bottom-up way on the properties, in particular the salience, of the input signal itself and (ii) on top-down modulation of the canonical microcircuit by the global network. The selection of input according to its salience, that is its prominence in terms of magnitude and duration, is for example associated with steering visual attention [10], selective reaction to sensory input, and determination of processing pathways [11].

For processing temporally structured information a fast working memory mechanism is required that does not rely on structural (e.g., synaptic) changes [12]. Bistable dynamics in a canonical microcircuit is one possible realization of such a mechanism.

In contrast to other parts of the brain, such as brain stem, cerebellum or thalamus, the circuitry of the cortex is mainly characterized by recurrent excitatory and inhibitory feedback loops at the local level, and by bidirectional sparse excitatory connectivity at the global level [13]. A model of a local cortical microcircuit should therefore feature pyramidal cells with long axons projecting to distant cortical areas, as well as local excitatory and inhibitory feedback loops. Such basic architectures, found at various spatial scales, have been represented in the parsimonious form of neural mass/field models [14–18]. Investigations of the steady-state behavior of such models have demonstrated that they may indeed provide the foundations for the aforementioned basic operations by featuring bistability and bifurcations [19–21]. Here, we extend those findings to the responses to transient stimuli and thereby gauge the implementation of signal gating and working memory operations. In this context, we investigate the influence of the following structural and physiological issues that have not been studied before in neural mass models of canonical microcircuits:

- **Indirect versus direct excitatory feedback.** Most neural mass and field models only consider a single excitatory neural population [e.g., 17], where the excitatory feedback loop is modeled as a recurrent (direct) feedback. Other approaches [e.g., 16] distinguish between pyramidal cells, which provide long-distance output connectivity, and excitatory interneurons, which are the main receivers of bottom-up input [2, 22]. In these three-population models the excitatory feedback to the pyramidal cells is indirect, mediated by the excitatory interneurons. Garnier and colleagues [23] have examined the consequences of direct versus indirect excitatory feedback and reported that an indirect feedback path provided additional dynamics. However, the relevance of these additional dynamics needs to be balanced against the costs of increased model complexity and should be evaluated with respect to the specific modeling requirements [2]. Therefore, we assess the sensitivity of the basic operations of a canonical microcircuit with respect to this choice.
- **Recurrent inhibitory feedback.** The axons of inhibitory interneurons form collaterals that target the same or other inhibitory neurons. This ‘inhibition of inhibition’ is incorporated into some models [17], while in others it is disregarded [16]. The nonlinear effect of this disinhibition with respect to the basic microcircuit operations will be investigated by numerical simulations.
- **Local network balance.** It has been shown that the relationship between inhibition and excitation in a neural assembly is of central importance for its information processing capacity [24]. In consequence, it mediates higher-order brain functionality [25] and its disruption disturbs cortical processing mechanisms and can lead to severe brain malfunctions and disorders, such as epilepsy [26, 27], autism [28–30], schizophrenia [31, 32], and excitotoxicity [33]. The healthy brain automatically establishes a dynamic balance of excitation and inhibition. This has been shown theoretically [34] and experimentally in both *in vitro* [35] and *in vivo* studies [26, 36]. As part of this study it is therefore investigated how the local network balance influences the functional capabilities of local microcircuits and how the canonical microcircuits can be individualized.

Networks composed of multiple canonical microcircuits realized by neural mass models have been recently used to explain experimental data in neurocognitive experiments, notably within the framework of *dynamic causal modeling* (DCM) [37, 38]. However, little attention has been devoted to two aspects: (i) the relationship between the network behavior and the intrinsic properties of the microcircuits, and (ii) the mechanistic explanation of behavior, rather than brain imaging data. Here we describe, as a simple example, a sentence processing network consisting of canonical microcircuits, which is flexible in processing the arrangement of words and enables the differentiation between alternative interpretations of ambiguous sentences. We show that the precise tuning of the local network balance is critical for the functioning of the proposed sentence processing model.

## Methods

### Description of the canonical neural population model

**Fig 1:**
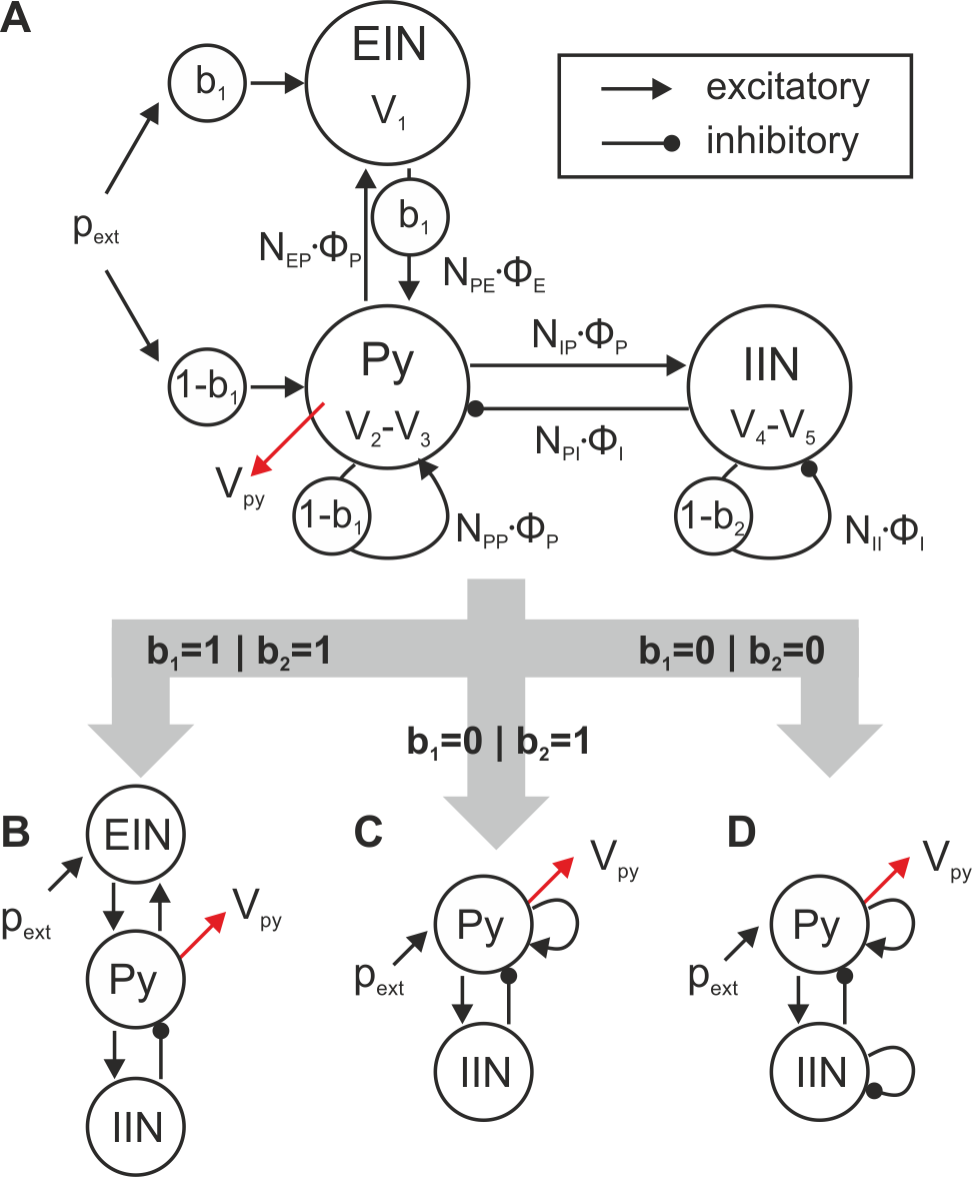
Generalized architecture of the neural mass model. A) The neural mass model accounts for excitatory interneurons (EIN), inhibitory interneurons (IIN), and pyramidal cells (Py). The architectural parameter b_1_ controls the deployment of direct and indirect excitatory feedback as well as the input receiving population, whereas the consideration of inhibitory collaterals is governed by the architectural parameter b_2_. This parameterization allows for a comparative investigation of relevant changes in the dynamical behavior among the three distinct architectures: B) a three-population model, C) a two-population model, and D) a two-population model with recurrent inhibitory feedback of the IIN. The transmitted mean firing rates φ(t) are scaled by connectivity gains N_ab_ between the source population, *b*, and the targeted population, *a*, respectively. The membrane potential of the pyramidal cells, V_py_(t) = V_2_(t)-V_3_(t), represents the output of the model (indicated by red arrows), detectable, for example, by EEG.

In the following, we present the neural mass model which is employed in this study. Neural mass models are an established approach to explain electroencephalography data [16, 38, 39], elucidate epileptogenic processes [40, 41] and electrical brain stimulation [42, 43], and investigate the dynamical behavior of a circumscribed neural area [19–21]. In this study, we employ a neural mass model that has three neural masses, or populations, representing the pyramidal cells (Py), excitatory interneurons (EIN), and inhibitory interneurons (IIN). The two interneuron populations form feedback loops on the Py (Fig 1A). Each of these neural masses is described by the mean membrane potential V(t), which is coupled to the mean firing rate φ(t) of the population through a non-linear activation function S(V(t)). For the definition and parameterization of the synaptic response and the activation functions we follow the approach by Spiegler [20], which is based on earlier descriptions [16, 39]. In each neural mass the afferent mean firing rate φ(t), arriving at the dendritic tree of a neural population, is transformed to a respective mean membrane potential V(t) by convolving the firing rate with a synaptic response kernel h_e,i_(t) as in

$$V(t)=\varphi (t)*h_{e,i}(t),$$ (1)

where the index *e* (*i*) denotes the synaptic response kernel of an excitatory (inhibitory) neural mass. The synaptic response kernel is modeled as an alpha-function

he,i(t)=He,iτe,i∙t∙θ(t)∙e−tτe,i, (2)

where θ(t) denotes the Heaviside function, H_e,i_ is the synaptic gain, reflecting number and efficacy of synaptic contacts, and *τ*_*e*,*i*_ is the characteristic time constant of either excitatory or inhibitory operating neural masses. The mean membrane potential V_c_(t), *c* ∈ [P,E,I], of the respective neural masses then depends on the sum of all incoming input components. Using Green’s function this can be expressed as:

$$D_{e,i}V_{c}=\sum \varphi_{in}(t),$$ (3)

where *D* is a second order temporal differential operator, which reads

$$D_{e,i}=\frac{\tau_{e,i}}{H_{e,i}}\left(\frac{d^{2}}{{dt}^{2}}+\frac{2}{\tau_{e,i}}∙\frac{d}{dt}+\frac{1}{{\tau_{e,i}}^{2}}\right),$$ (4)

where Eq (2) represents the Green’s function of this differential operator. This operator is then decoupled into two first-order differential equations. The transformation of mean membrane potential to mean firing rates, representing the processes occurring at the axonal hillock of a neuron, are modeled by a sigmoidal activation function, in this case the logistic function

$$\varphi (t)=S(V_{c}(t))=\frac{2e_{o}}{1+e^{r(v_{0}-V_{c}(t))}}.$$ (5)

While *e*_*0*_ represents the half of the highest achievable firing rate, *r* is the maximum slope of the sigmoid function and *v*_*0*_ denotes the membrane potential for which half of the maximum firing rate is invoked. The mean membrane potential of the Py, integrating both positive and negative feedback, forms the observable signal of the circuit (e.g., by EEG) and, at the same time, gives rise to the output signal to distant areas through the activation function. Hence, the description of observable dynamics is centered on this principal cell population.

We construct a general model formalism that accounts for distinct local feedback topologies: (i) a three-population model with an indirect excitatory feedback path through the EIN (Fig 1B), (ii) a two-population model with direct excitatory feedback through self-connections of the Py (Fig 1C), and (iii) a two-population model with direct excitatory feedback and recurrent inhibitory feedback of the IIN (Fig 1D). The local topologies are controlled by two parameters b_1_ and b_2_. The first parameter (b_1_) allows a gradual transition between the two-population models, with extrinsic input, p_ext_, received by the excitatory population (Py), and the three-population model with extrinsic input, p_ext_, received by the excitatory interneurons (EIN). Importantly, intermediate situations can be modeled, where both excitatory populations receive extrinsic input and, both, direct and indirect excitatory feedback loops co-exist (0< b_1_ <1). This approach enables the treatment of a principle choice in model structure (two vs. three-population model) as a continuous parameter, which can be subjected to, for example, bifurcation analysis. See S1 File for more details on the mapping of the three to the two-population model. The second parameter (b_2_) controls the presence of the recurrent feedback loop for the IINs. According to the scheme depicted in Fig 1A, the system of governing equations of the canonical microcircuit reads:

$$\begin{array}{l} D_{e}\cdot V_{1}=N_{EP}\cdot \varphi_{P}+b_{1}\cdot p_{ext}\\ D_{e}\cdot V_{2}=b_{1}\cdot N_{PE}\cdot \varphi_{E}+\left(1-b_{1}\right)\cdot N_{PP}\cdot \varphi_{P}+\left(1-b_{1}\right)\cdot p_{ext}\\ D_{i}\cdot V_{3}=N_{PI}\cdot \varphi_{I}\\ D_{e}\cdot V_{4}=N_{IP}\cdot \varphi_{P}\\ D_{i}\cdot V_{5}=\left(1-b_{2}\right)\cdot N_{II}\cdot \varphi_{I}\\ \;\varphi_{P}=S\left(V_{Py}\right)=S\left(V_{2}-V_{3}\right)\\ \;\varphi_{E}=S\left(V_{E}\right)=S\left(V_{1}\right)\\ \;\varphi_{I}=S\left(V_{I}\right)=S\left(V_{4}-V_{5}\right) \end{array}.$$ (6)

The parameters N_ab_ denote the connectivity gains between the source population *b* and the target population *a*, where *a*,*b* ∈ [P,E,I]. For the numerical integration of this system of non-linearly coupled linear ordinary differential equations Heun’s method is employed. The operators *D*_*e*,*i*_ and *S(∙)* denote the excitatory (inhibitory) temporal differential operator and the sigmoidal activation function, respectively (see Eqs (4) and (5)). The stability of the integration for the integration interval of 1ms was checked. The system is initially parameterized according to a previously used configuration [16, 39], see Table 1. Further, the connectivity gains N_PP_ and N_II_ are determined to be N_PP_ = 113.4 (see S1 File) and N_II_ = 33.25, similar to other inhibitory connection strengths.

**Table 1:** Parameterization of the employed neural mass model.

Parameter	Value	Unit	Parameter	Value	Unit	Parameter	Value	Unit
H_e_	3.25	mV	N_PE_	0.8*N_EP_	-	r	0.56	mV^-1^
H_i_	22	mV	N_IP_	0.25*N_EP_	-	v_0_	6	mV
τ_e_	10	ms	N_PI_	0.25*N_EP_	-	e_0_	2.5	s^-1^
τ_i_	20	ms	N_PP_	113.4	-			
N_EP_	135	-	N_II_	33.25	-			

### Definition and parameterization of network balance

The relationship of inhibition and excitation, often referred to as network balance, in a neural assembly regulates the interaction of neural units, affects the dynamics of brain states, and is associated with severe brain disorders, such as epilepsy [26, 27], autism [28–30], or schizophrenia [31, 32]. The concept of network balance is ambiguous and difficult to quantify simply as ratio of excitation and inhibition in a neural system. This is due to the multiple spatial and temporal scales in the brain [24, 28] and the multiple structural and functional aspects that could be considered. A description of network balance on a mesoscopic level of interacting neural populations may focus on structural influences, such as topology, number, and efficacy of synaptic contacts, or functional features, such as conveyed firing rates, or factors of the synaptic response. In computational models of other studies, proposed approaches relate excitatory and inhibitory charges, conductances [27, 28, 36], or membrane potentials [24] to each other. Often the network balance is defined in a network context as the ratio of recurrent inhibition to excitation [44, 45]. However, this is limited to two population models and becomes ambiguous for multiple population models such as those used in our study.

In our model, the pertinent parameters that are potentially relevant for excitation and inhibition include: (i) the synaptic response function (time constants, synaptic gains), (ii) the external input to all three populations, (iii) the parameters of the sigmoidal activation function, and (iv) the connectivity gains between the populations. Among these parameters, the synaptic gains H_e,i_, the connectivity gains, i.e. N_EP_, N_PE_, N_PI_, N_IP_ N_PP_, and N_II_, and the external inputs have the most direct and biologically plausible effect on the excitation and inhibition of the system. However, note the formal redundancy, yet conceptual difference, among synaptic gains and connectivity gains in the system equations. According to the governing model equations, i.e. Eqs (1)–(6), H_i_, N_PI_, and N_II_ reflect a gain of inhibitory feedback just with different scaling. Also, varying H_e_ is equivalent to synchronously varying N_EP_, N_PE_, N_IP_, and N_PP_. Thus, in the interest of tractability, for the investigation of the modulating influence of excitation and inhibition to the local dynamics, consideration of a parsimonious set of parameters is sufficient. Hence, we focus our analysis on the influence of H_e_ and H_i_, which are interpreted to represent efficacy and density of excitatory, e.g. AMPA, and inhibitory, e.g. GABA_A_, neurotransmitter receptors. This is equivalent to the number and strength of the synaptic weights.

### Bifurcation analysis, simulations, and dynamic function map

The model equations were simulated in a dimensionless form in Matlab (The MathWorks, Inc., Natick, Massachusetts, USA) and a bifurcation analysis was performed using the numerical continuation tool DDE-BIFTOOL [46]. Standard methods to compute fixed point curves were used, i.e. computation of fixed points, derivation of Jacobian matrix, linearization of the system around the fixed points, and evaluation of the eigenvalues to determine the local stability. The synaptic gains H_e,i_ served as bifurcation parameters in the respective local topologies. The simulations were of 5 seconds length and the state variables were initialized with a zero vector. Due to the initialization of the system with an external input level of p_ext_ = 0s^-1^, the system consistently resided on the lower branch of the S-shaped fixed point curve in the case of a bistable regime.

**Fig 2:**
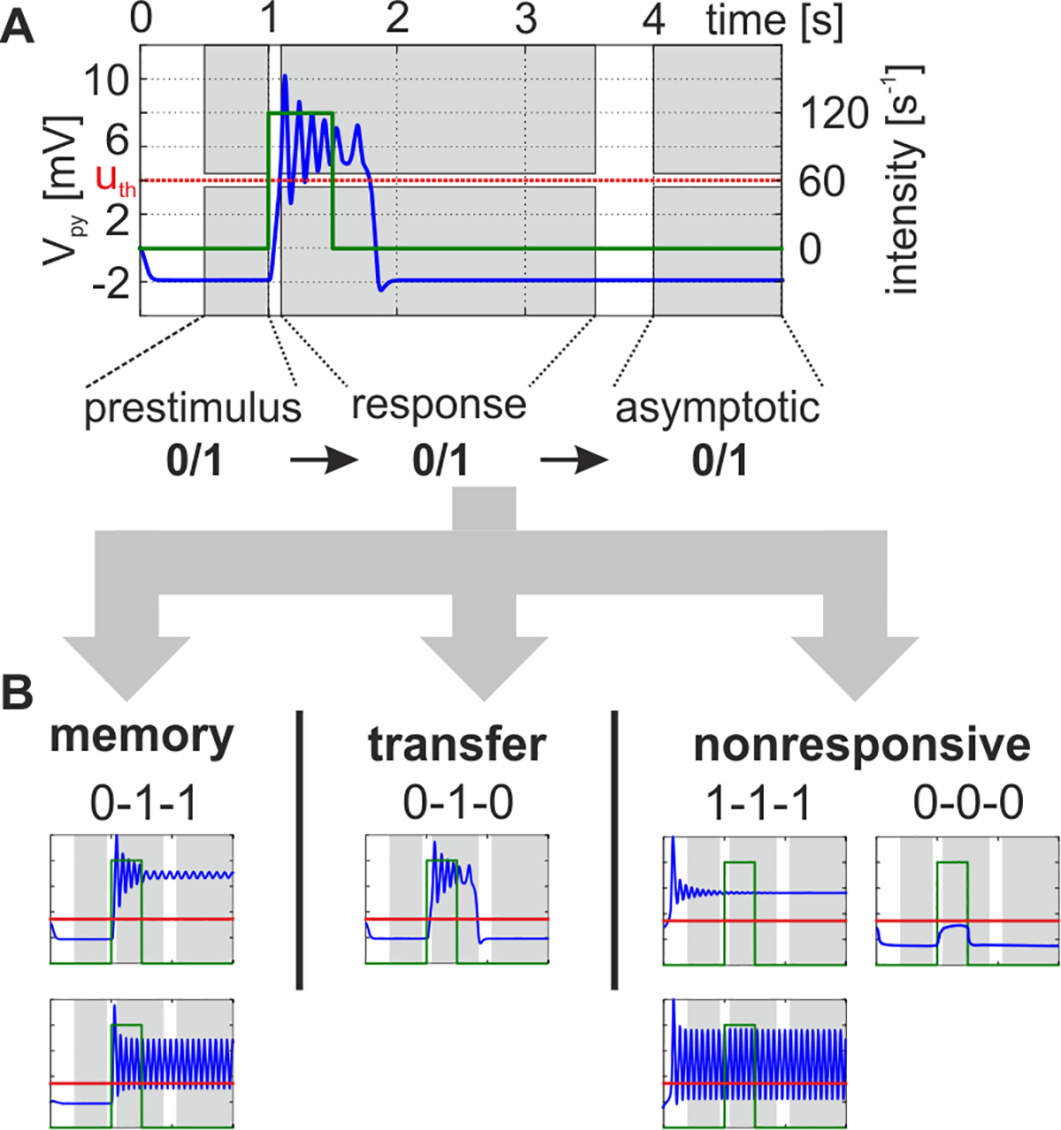
Stimulation principle and categorization of the dynamic response behavior. A) The model received a rectangular stimulation of varying intensity and duration (green line). The maximum of the mean membrane potential of the pyramidal cell population (blue line) was recorded in three time windows, i.e. prestimulus window, immediate response window, and the asymptotic window (gray shaded areas). To classify the response behavior, these activation values were compared to a threshold of 4mV (red horizontal line, u_th_) in each window–‘0’ denoting a subthreshold activation and ‘1’ denoting an activation exceeding the threshold. B) The combined evaluation of the activities (e.g., ‘0-1-1’) led to three distinct classes of response behaviors: memory, transfer, and nonresponsive behavior. For the plotted curves we used b_1_ = 1, b_2_ = 1, H_e_ = 3.25mV, and H_i_ = 22mV.

In each simulation, the model was stimulated with a rectangular impulse of defined intensity, ranging between 50s^-1^ and 250s^-1^, and duration, ranging between 500ms and 1500ms, starting after a 1s settling time. The dynamic response behavior of the canonical microcircuit to this stimulation was categorized within three different time windows, through comparison of the maximum membrane potentials of the pyramidal cell population with a firing threshold. The time windows were: i) the prestimulus window (0.5s–1s), ii) the immediate response window (1.1s to 3.5s), and iii) the asymptotic window (4s to 5s), see Fig 2A. The firing threshold u_th_ = 4mV was defined relative to the maximum firing rate of 5s^-1^, so that about 25% of the maximum firing rate is reached at the threshold. In each time window the system was considered to be active if the maximum activation exceeded the threshold and to be inactive if the maximum activation was below the threshold. Three general types of behavior were observed (see Fig 2B): i) a memory behavior, when the system remains permanently active after the input has ceased, ii) a transfer behavior, when activity is above threshold during the immediate response window but below otherwise, and iii) a nonresponsive behavior, when the maximum activity is consistently above or below the threshold in all three windows. Note that in some cases the activity oscillates around the threshold. In these cases the population can activate postsynaptic populations at least for part of the time and is therefore considered to be above threshold. The occurrence of these behaviors in dependence on the governing parameters is summarized in so-called *dynamic function maps*, which typify the dynamical response repertoire of the respective parameterization.

## Model evaluation

In this section we describe the evaluation of the proposed canonical microcircuit model with respect to (i) the consideration of indirect or direct excitatory feedback, (ii) recurrent feedback to IIN, and (iii) the local network balance, by means of bifurcation plots and dynamic function maps. We show that, and under which conditions, the model supports mechanisms for signal flow gating and working memory.

### Principal dynamics

**Fig 3:**
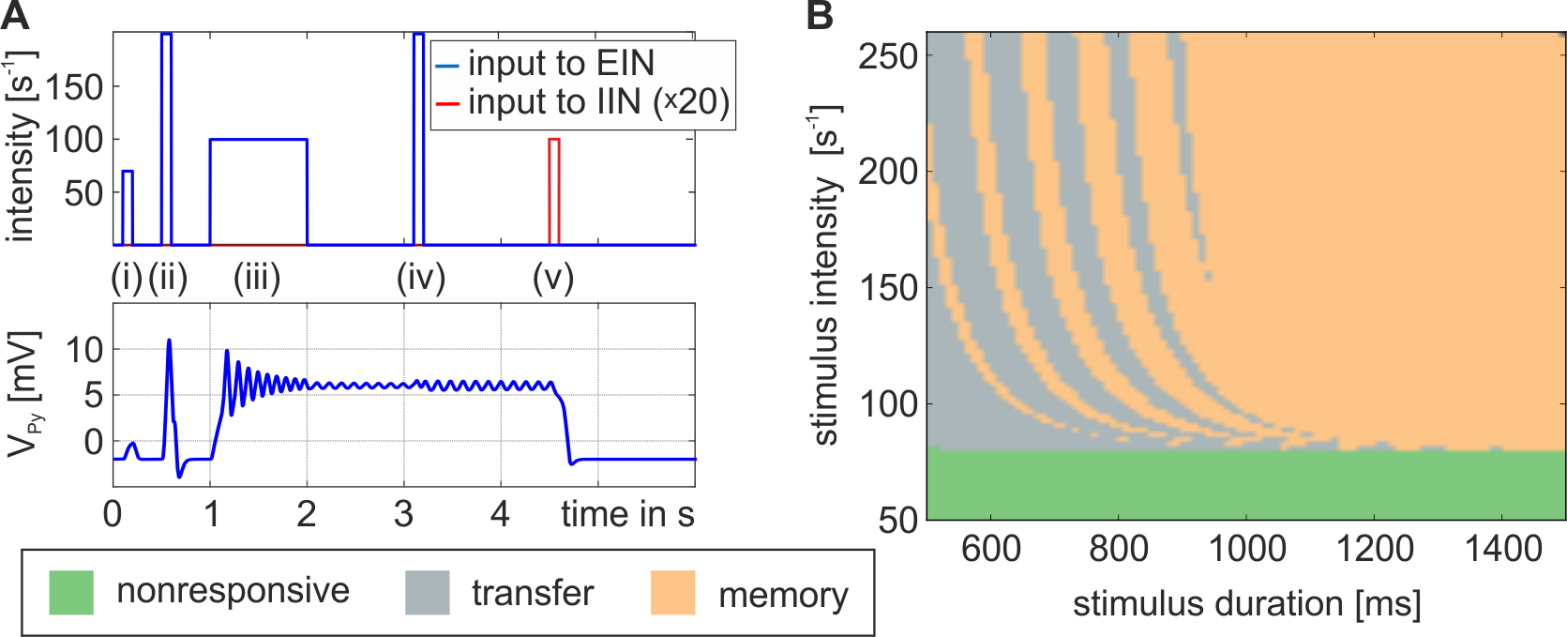
Aspects of the models’ responsiveness to afferent stimuli arriving at the EIN of the three population model. A) Depending on the salience of the applied stimuli in terms of duration and intensity, three distinct response behaviors were observed: (1) a nonresponsive behavior following weak and brief stimulation, where the Py’s membrane potential, V_Py_, responds only with a small deflection below a firing threshold, see impulse (i), (2) a transfer behavior following a strong and brief stimulation, where V_Py_ exceeds a firing threshold, see impulse (ii), and (3) a memory behavior following longer stimulation of medium intensity, see impulse (iii), for which the system can settle down on a stable state of higher activation. In this state the system is insensitive to further stimuli, or noise, (see impulse (iv)), but can be actively reset through a weak and brief impulse to the IIN, clearing the memory trace, see impulse (v). Please note that this IIN impulse was enlarged by a factor of 20 to improve clarity. B) The response behaviors depend on the salience of the input. A nonresponsive behavior is shown for intensities below 78s^-1^ (green region). Exceeding this intensity, a longer stimulus is able to reliably evoke the memory behavior (orange region). The shorter the stimulus the more likely the transfer behavior (grey region) becomes, where the stripe-like patterns signify a dependency of the behavior (transfer or memory) on the phase relation between stimulus switch-off time and the intrinsic system oscillation. For the plotted curves we used b_1_ = 1, b_2_ = 1, H_e_ = 3.25mV, and H_i_ = 22mV.

In the following we describe the key features of the gating mechanism for the three-population model, which features indirect excitatory feedback (Fig 1B). This configuration considers separate neural masses for input and output. Rectangular bursts with a distinct intensity and duration were applied to the excitatory interneuron population (EIN). These bursts mimicked input from upstream sources, such as sensory information stemming from primary cortical areas, or higher level information, such as spoken words or phonemes. Fig 3A summarizes the distinct response behaviors: The system responds to weak and brief stimuli with a small deflection of the Py membrane potential (nonresponsive behavior), but to stronger, though still brief, stimuli with a large transient, exceeding the firing threshold of 4mV (transfer behavior). In both cases the system settles down to its original state shortly after the stimulus is turned off. In contrast, for longer lasting stimuli the system settles down in a stable state of higher activation and remains insensitive to further stimuli or noise (memory behavior). Being in this stimulus-selective high-activity state, a brief input to the IIN can actively reset the system to the lower activated state (S2 Fig). Whether the response is nonresponsive, transfer, or memory, depends on the salience of the applied stimulus in terms of intensity and duration, see Fig 3B. The diagram in Fig 3B serves as a characteristic fingerprint and maps the observable response dynamics. The respective basic operations of gating and storage may serve as building blocks for more complex mechanisms like decision-making, based on neural interaction in a single neural area. The stripe-like patterns in the transition zone between areas of transfer and memory behavior in Fig 3B signify a dependence on the stimulus switch-off time relative to the phase of the system’s intrinsic oscillations (see S3 Fig).

**Fig 4:**
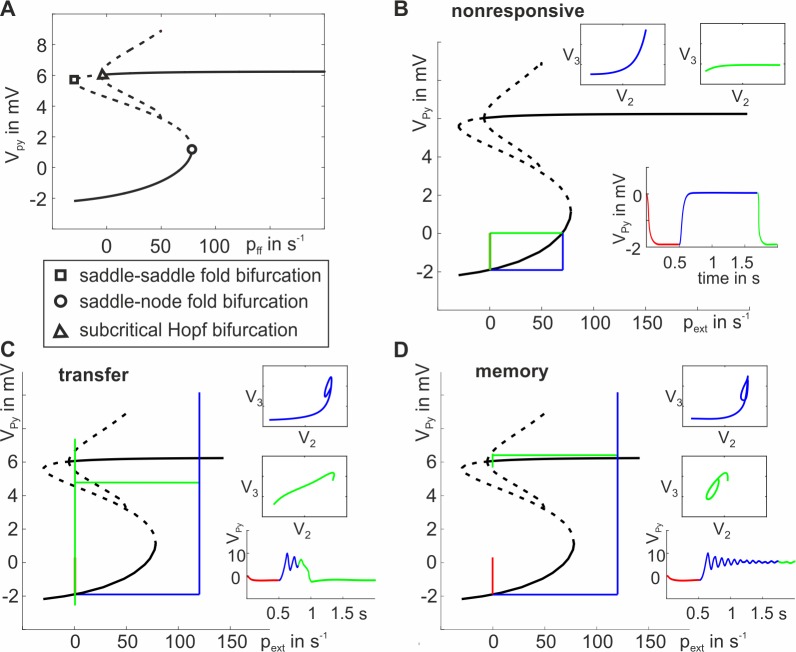
Dynamics of the distinct response behaviors in a projection of the state space. A) The S-shaped fixed point curve features stable (solid line) and unstable (dashed line) fixed points for varying input strengths to the EIN. Two fold bifurcations (saddle-node and saddle-saddle) and a subcritical Hopf bifurcation were identified. B-D) Projections of the response behaviors in the bifurcation diagrams with inlets illustrating state space trajectories and the respective time courses: nonresponsive (B), transfer (C), and memory (D) behavior. Note that V_Py_(t) = V_2_(t)-V_3_(t). Color-coding distinguishes prestimulus (red), response (blue), and asymptotic (green) mean membrane potentials.

When looking on the underlying structure of the state space, it becomes apparent that the observed behavior is based on a rather simple mechanism. In Fig 4A, the fixed point curve of the steady state behavior of the three-population model with default parameterization is visualized. A fold bifurcation was identified at each turning point of the fixed point curve–one of saddle-node type (unstable-stable) and one of saddle-saddle type (unstable-unstable). Further, a subcritical Hopf bifurcation is identified at p_ext_ = -5.9s^-1^. The resulting separatrix marks an unstable manifold, which repels local trajectories in the state space close to it. If no input is fed into the system (p_ext_ = 0s^-1^), the system resides on the lower branch of the fixed point curve with V_Py_ ≈ -2mV, see Fig 4A. If a weak impulse (p_ext_<78s^-1^, see Fig 4B) is applied to the EIN, the system is not able to pass the lower fold bifurcation and settles down on the lower branch of the fixed point curve again. If the input p_ext_>78s^-1^, the system passes the lower fold bifurcation and settles on the upper branch of the fixed point curve, thus exceeding the firing threshold of 4mV. The existence of a pair of negative conjugate complex eigenvalues leads to a damped oscillation. When the stimulus is switched off, the system’s input returns to its original value (p_ext_ = 0s^-1^). If the system’s trajectory is located outside the Hopf separatrix (i.e. the system was not damped sufficiently) the system settles down at the lower branch of the fixed point curve, realizing the transfer behavior (Fig 4C). If, however, the system’s trajectory is located within the Hopf separatrix (i.e. the oscillations has damped sufficiently due to ample settling time), the system settles on the upper branch of the fixed point curve, thus showing the memory behavior (Fig 4D). In summary, the hallmarks of the described mechanism are (i) bistable activation of the Py population (high and low state), (ii) selectivity for salient stimuli, (iii) reduced sensitivity to further stimuli in the high state, (iv) relative robustness to noisy fluctuations in external input in each state, and (v) a phase-dependency of the stimulus offset, where certain phases allow the system to settle down in the high activated state while other phases do not (see S3 Fig for more information).

In the following subsections, we will assess the effects of the positive and negative feedback structures to the described response behavior. For this purpose, we varied the excitatory and inhibitory synaptic gains H_e,i_ (controlling the network balance). For each variation, the characteristic fingerprint (see Fig 3B) was generated and qualified in the state space. We evaluated three distinct architectures with respect to the local network balance: (i) indirect excitatory feedback architecture with no recurrent IIN self-feedback (Fig 1B), (ii) direct excitatory feedback architecture with no recurrent IIN self-feedback (Fig 1C), and (iii) direct excitatory feedback architecture with recurrent IIN self-feedback (Fig 1D).

### Indirect excitatory feedback architecture

**Fig 5:**
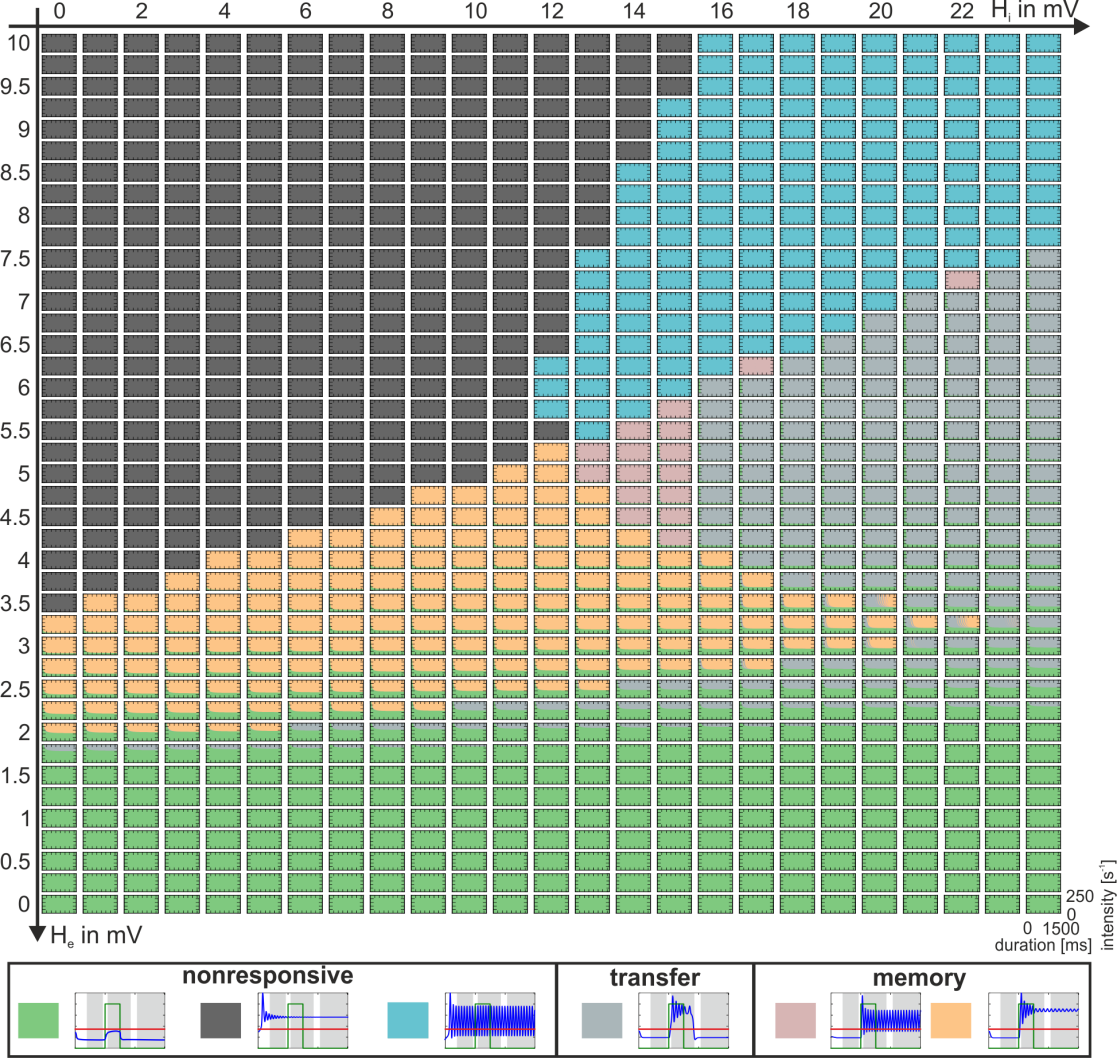
Dynamic function map for the indirect excitatory feedback architecture (see Fig 1B). Collection of characteristic fingerprints for varying excitatory (H_e_) and inhibitory (H_i_) synaptic gains. Colors code the observed response behaviors: nonresponsive (bright green, anthracite and cyan regions), transfer (grey regions), and memory (orange and rose regions). The local network balance controls the dominance of the behaviors and tunes the criticality of the system. See S4 Fig for a duplication of this figure, extended by explanatory state space diagrams.

If both parameters *b*_*1*_ and *b*_*2*_ are set to one, the excitatory feedback loop becomes purely indirect (i.e., the output of the Py is fed into the EINs, which in turn project back to the Py) and the recurrent IIN self-feedback loop disappears (i.e., the three-population model, see Fig 1B). Fig 5 shows the dynamic function map, a collection of characteristic fingerprints (Fig 3B). The dynamic function map charts the classified response behaviors in the parameter space spanned by H_e_ and H_i_ and reveals regions where the system is dominated by the nonresponsive (bright green, anthracite and cyan regions), transfer (grey regions), and memory behaviors (orange and rose regions) as well as compositions thereof. The default ratio of excitation and inhibition (H_e_ = 3.25mV and H_i_ = 22mV) [16], denoting the network balance, is located just at the tip of a larger memory-dominated region (orange region) corresponding to strong inhibitions levels, and close to a region dominated by transfer behavior. Such proximity of a system’s state to major transition zones of the system’s behavior (i.e., bifurcations) has been referred to as criticality and is considered beneficial for the system’s information processing capacity, as small parameters changes may produce large changes in behavior [47, 48]. Hence, the local network balance constitutes a very sensitive determinant of the canonical microcircuit’s behavior in that it tunes criticality. Further, the local network balance controls the perceptual sensitivity of the system concerning the intensity of afferent stimuli which are perceived or not by tuning the distance between the working point and the lower fold bifurcation (compare, for example, S5G and S5H Fig). In the default parameterization (H_e_ = 3.25mV and H_i_ = 22mV) this threshold was about p_ext_ = 78s^-1^. The threshold is raised when H_e_ is decreased, meaning a reduced sensitivity to external stimuli. In turn, the sensitivity is increased when this threshold is lowered through an increase of H_e_. Note, however, that a pure increase of H_e_ would result in a transfer dominated behavior whereas a sensitivity increase in favor of memory behavior demands a simultaneous decrease in inhibition (Fig 5).

**Fig 6:**
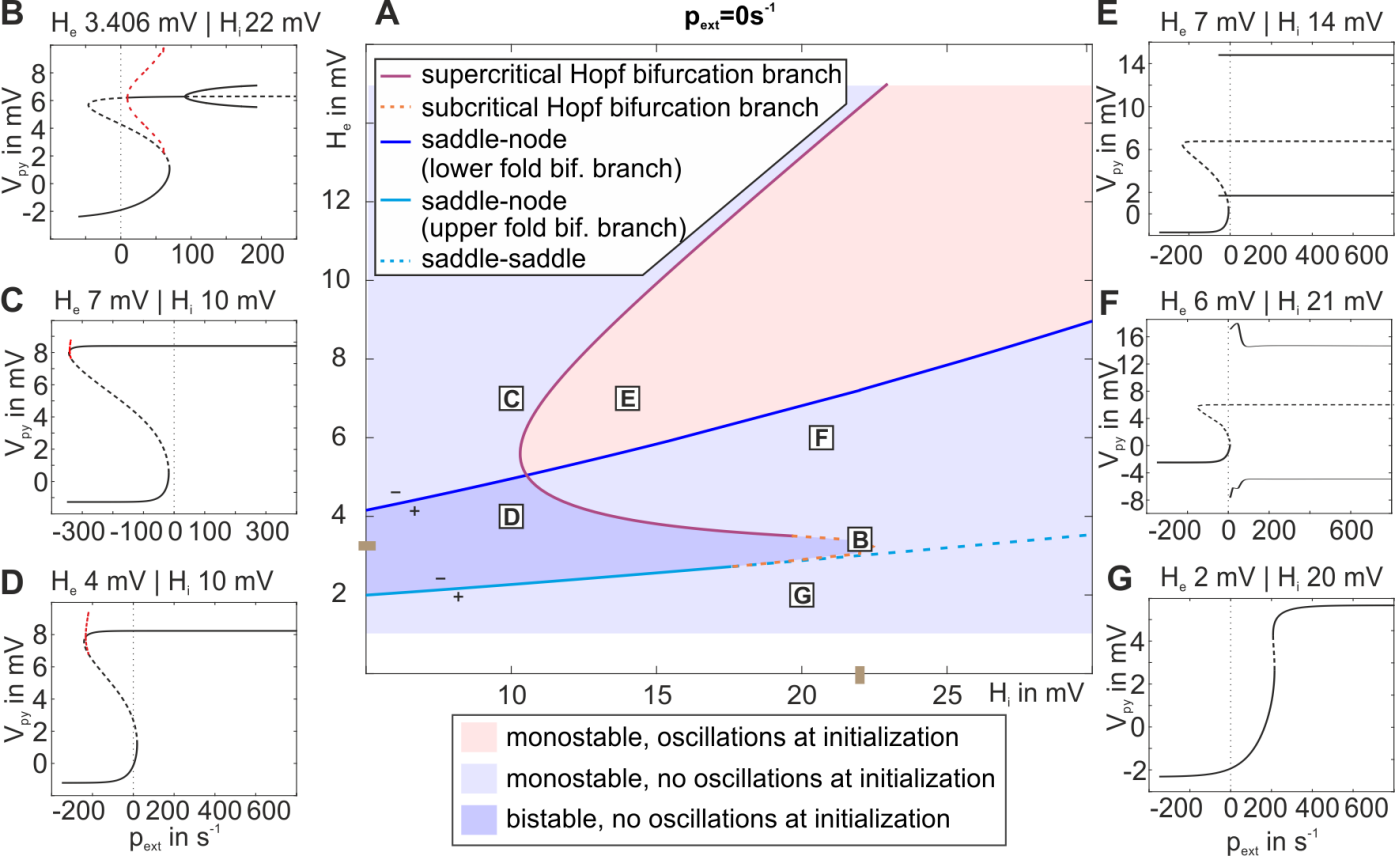
Two parameter bifurcation plot of the three-population model. A) The plot characterizes the existing bifurcations (with respect to p_ext_) at p_ext_ = 0 for the indirect excitatory feedback architecture and tracks them through the parameter space spanned by the excitatory and inhibitory synaptic gains H_e_ and H_i_. The background is colored light red for oscillating behavior in the low state at p_ext_ = 0s^-1^, light blue for non-oscillatory behavior and monostability, and dark blue for no oscillations and bistability. Brown marks at the axis denote default parameter values for H_e_ and H_i_. The region between the upper (cyan line) and lower (blue line) fold bifurcations and the Hopf bifurcation (purple/orange curve) exhibits bistability, where memory behavior is possible and H_e_ and H_i_ tune robustness and sensitivity of the system. Signs + and–indicate whether the particular fold bifurcation is located at positive or negative values relative to the working point. The solid purple line denotes the supercritical Hopf bifurcation branch. The dashed orange line denotes the subcritical Hopf bifurcation branch, which is important for the transfer behavior of the system. Together, the branches mark the border between dominant regions of memory and transfer behavior (compare S4 Fig). B)-G) Bifurcation diagrams characterizing stable and unstable fixed points for a broad range of input values.

In order to better understand the mechanism behind how the network balance changes the response behaviors we further characterized the system at its working point p_ext_ = 0. We kept the external input at zero, systematically varied H_e_ and H_i_, and tracked the relevant bifurcations, as shown in Fig 6A. The background of the plot is colored light red for oscillating behavior in the low state at p_ext_ = 0s^-1^, light blue for non-oscillatory behavior and monostability, and dark blue for no oscillations and bistability. The default parameter values for H_e_ and H_i_ are indicated on the axes. Note that the emerging graphs strikingly reflect the borders of the response behavior regions from Fig 5. The blue line in Fig 6A indicates the lower fold bifurcation for p_ext_ = 0. Below that line this bifurcation will be located at p_ext_>0 and above it at p_ext_<0 with respect to p_ext_, compare Fig 6D and 6C. Likewise the cyan line indicates the upper fold bifurcation for p_ext_ = 0s^-1^, which is located at p_ext_<0 above that line and at p_ext_>0 below the line, compare Fig 6D and 6G. In consequence, only for the area between the two fold bifurcation branches, the point p_ext_ = 0s^-1^ will be located between the two fold bifurcations–a necessary condition for bistability at that point. For the memory behavior, it is a necessary prerequisite that bistability exists without input. Therefore, the two fold bifurcation lines delimit the area in the H_e_-H_i_ plane, where memory behavior is possible. This is in agreement to the observed behavior shown in Fig 5. Further, between those lines the distance between the upper and lower fold bifurcations on the p_ext_ axis (see Fig 6B–6G) determines the robustness of the system to noise, by scaling the width of the bistability. Moreover, the system’s location relative to the lower fold bifurcation determines its sensitivity to stimuli. However, not the entire area between the two fold bifurcation branches actually exhibits bistability. This is because at some point, when H_i_ is increased, the upper fold bifurcation, switching between unstable and stable fixed points (saddle-node bifurcation, solid cyan line in Fig 6A), separates into a fold bifurcation between two unstable fixed points (saddle-saddle bifurcation, dashed cyan line), and a subcritical Hopf bifurcation, indicated by the dashed orange curve. To the left of that curve the subcritical Hopf bifurcation occurs at p_ext_<0 leading to bistability for p_ext_ = 0 in form of a stable focus on the upper branch of the fixed point curve, while to the right of that curve the Hopf bifurcation is at p_ext_>0. In consequence to the latter, the upper branch of the fixed point curve is unstable for p_ext_ = 0 and bistability is abolished (Fig 6B). At some point along this subcritical Hopf bifurcation branch, the subcritical Hopf bifurcation turns into a supercritical Hopf bifurcation, indicated by the solid purple curve in Fig 6A. Instead of the stable focus, the stable limit cycle, associated with the supercritical Hopf bifurcation, then accounts for the bistability at p_ext_ = 0. In consequence, there is also bistability along a narrow strip on the right side of the supercritical Hopf bifurcation in Fig 6A, where the upper state is oscillatory.

Note that the mentioned subcritical and supercritical Hopf bifurcations collide at positive/negative values of p_ext_ within a small region close to the dashed/solid purple line, respectively (not shown here). This collision leads to a stable limit cycle, which either surrounds small bits of the overlapping part of the fixed point curve (see S5F Fig), giving rise to bistability and thus memory behavior, or reaches just up to the lower fold, abolishing bistability and leading to transfer behavior (see S5I Fig). In case of an overlap, the oscillations of this global limit cycle do cross the firing threshold (classified rose in S5F Fig) or do not cross the firing threshold (classified orange in S5G Fig), both indicating memory behavior. In summary, the dark blue area in the H_e_-H_i_ plane in Fig 6 exhibits bistability and allows for memory behavior, but also part of the light blue area (due to stable limit cycles), as corroborated by Fig 5. For a tuning of the network balance outside of this bistable region, the canonical microcircuit does not feature the memory behavior anymore and, thus, loses an integral part of the basic operations.

### Direct excitatory feedback architecture

As explained in the *Methods*, we may seamlessly transpose the indirect excitatory feedback architecture into a direct excitatory feedback one, without any qualitative change of the underlying cortical microarchitecture. For this two-population model the parameters equal b_1_ = 0 and b_2_ = 1 (see Fig 1C). The transition is constrained by conserving the total number of neurons in the system, the sum of the flowing currents and all connections between single neurons, as described in the S1 File. The two-population model does not divide the excitatory neurons into an input and an output layer. For a parameterization with default values for H_e_ and H_i_, no bistability was observed (see Fig 8A and 8E, at the working point there is only one stable fixed point).

**Fig 7:**
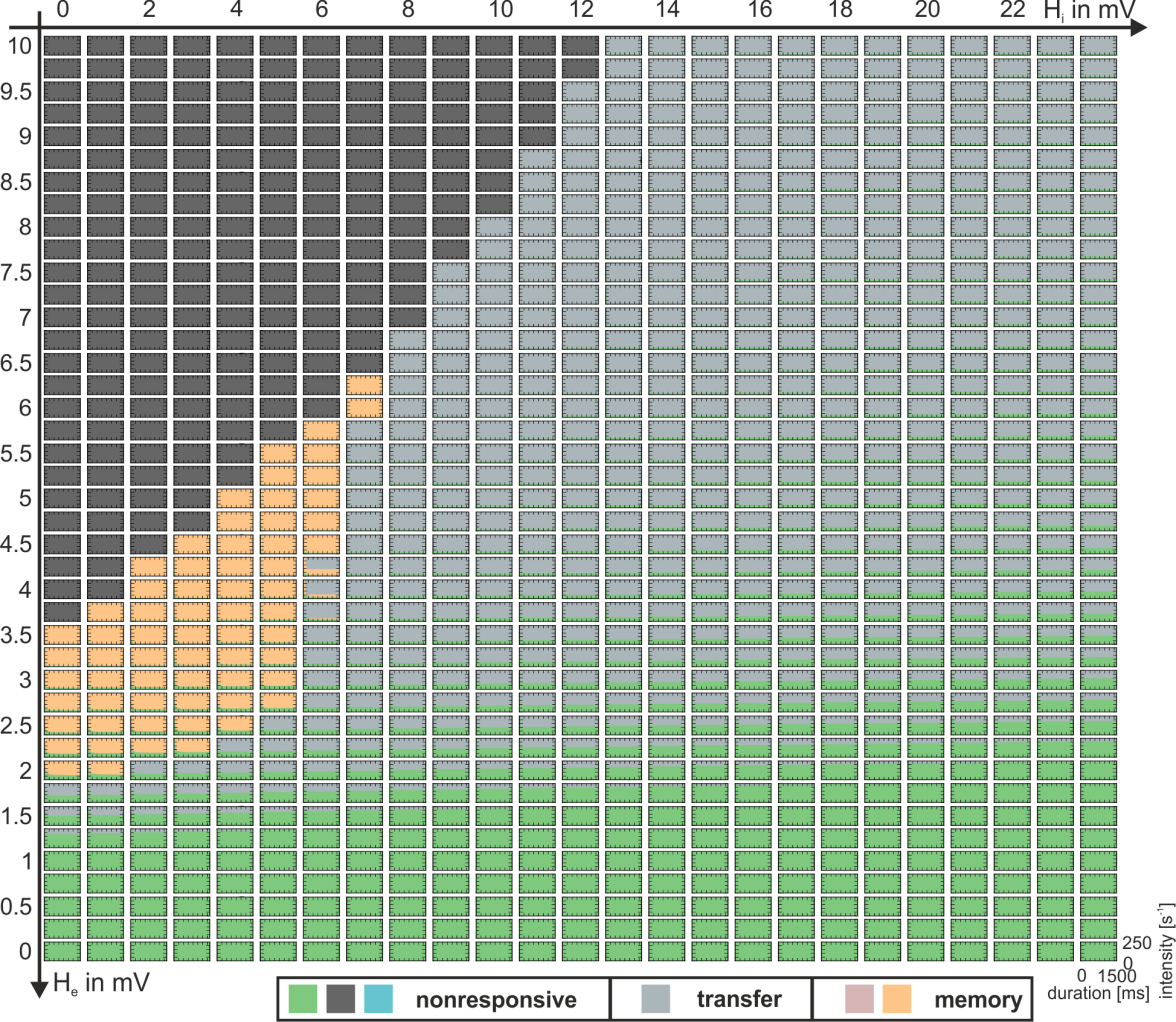
Dynamic function map for the direct excitatory feedback architecture (see Fig 1C). Collection of characteristic fingerprints for varying excitatory (H_e_) and inhibitory (H_i_) synaptic gains. Colors code the observed response behaviors: nonresponsive (bright green and anthracite), transfer (grey), and memory (orange). The variety of observed behaviors is reduced compared to the three-population case (Fig 5). However, all three main types are observable. See S5 Fig for a duplication of this figure, extended by explanatory state space diagrams.

**Fig 8:**
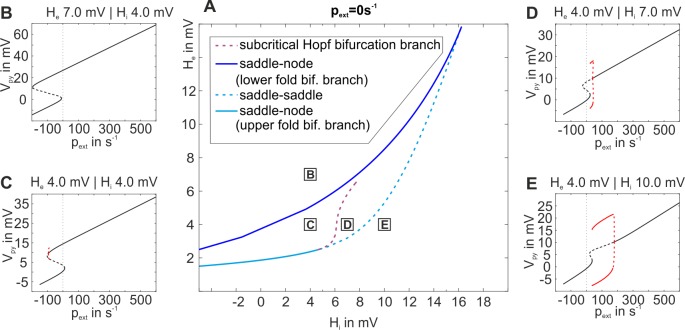
Two parameter bifurcation plot of the two-population model. The plot characterizes the existing bifurcations at p_ext_ = 0s^-1^ for the direct excitatory feedback architecture and tracks them through the parameter space spanned by the excitatory and inhibitory synaptic gains H_e_ and H_i_. The region between the upper (cyan line) and lower (blue line) fold bifurcation branch limits the region where a bistable fixed point curve is obtained. However, the subcritical Hopf bifurcation (purple line) renders parts of the fixed point curve instable and prevents an actual bistability at p_ext_ = 0s^-1^.This suppresses a memory behavior in favor of a transfer behavior (see Fig 7).

The dynamic function map for the direct excitatory feedback architecture was derived and is depicted in Fig 7. As no oscillatory behavior is observed (at the current working point p_ext_ = 0, see Fig 8), this map exhibits fewer variants as compared to the indirect feedback architecture (Fig 5), but still contains all types of the classified response behaviors, but for lower inhibitory synaptic gain. However, we could not find parameterizations where all three types of response behaviors are robustly present in a single fingerprint. Memory and nonresponsive behaviors still occur within single parameterizations and depend on the intensity but not on the duration of the stimulus anymore. Furthermore, the ability to respond with a damped oscillation to further input, being in the high state, is lost. Now, the system immediately settles down. Like in case of the indirect excitatory feedback, the system is in general more sensitive to changes in the excitatory synaptic gain than to those in the inhibitory synaptic gain. This is reflected by the respective ranges of synaptic gains necessary to obtain a desired response behavior.

As for the indirect excitatory feedback architecture, the existence of bifurcations was examined at the working point p_ext_ = 0s^-1^ and is depicted in Fig 8A. Again, the bifurcation branches reflect the borders of the response behavior regions in the extended dynamic function map from Fig 7. As described for the indirect feedback architecture, bistability and memory behavior generally exist within the region between the upper and the lower fold bifurcation branches, as long as they reflect saddle-node bifurcations. However, also similar to the indirect feedback case, at about H_i_ = 6mV the upper fold bifurcation (cyan curve in Fig 8A) splits into a saddle-saddle and a subcritical Hopf bifurcation, and only to the left of that Hopf bifurcation bistability exists, compare Fig 8C and 8D. In general, the membrane potentials in the high state are quite high (>10mV) and close to the saturation threshold of the sigmoid function at about 14.8mV. The efferent firing rates of such high membrane potentials will, hence, lead to almost invariantly transmitted firing rates of 5s^-1^. In consequence, graduated efferent firing rates, like in case of the indirect excitatory feedback architecture, are rather exceptional.

In summary, the two-population model exhibits all types of response behaviors, but for lower ranges of the inhibitory synaptic gain. A concomitant existence of all types of response behaviors for a single value of the local network balance was not observed.

### Direct excitatory feedback architecture with disinhibition

An often neglected property of neural architectures is the existence of recurrent inhibitory collaterals, leading to inhibitory self-feedback and resulting in a disinhibition effect. While inhibition dampens an excited system, disinhibition reduces this damping and makes the inhibitory feedback path less effective. This promotes saturation of the excitatory feedback path and may lead to stationary dynamics, where the membrane potential of the Py can rise in linear dependence on the external input. This effect is even more amplified for high excitatory (increase in positive feedback) and inhibitory (increase of disinhibition) synaptic gains.

We introduced disinhibition to the two-population model by setting the architectural parameter b_2_ to zero while keeping b_1_ = 0 (see Fig 1D), slowly increased the disinhibition controlling connectivity gain N_II_ and tracked existing bifurcations in the state space for the two-population model (see S1 Fig). Since the dynamics did not change significantly for large levels of disinhibition, we fixed N_II_ to N_II_ = 33.25, which equals the level of inhibitory connections targeting excitatory populations.

The dynamic function map for the direct excitatory feedback architecture with inhibitory recurrent feedback was derived and is shown in Fig 7. Again, all types of the classified response behaviors were observed. For some parameterizations two behaviors were observed in dependence on the stimulus’ intensity. Again, the system was more sensitive, in terms of different response behaviors, to changes in the excitatory synaptic gain than to changes in the inhibitory synaptic gain. As before, bifurcations at the working point p_ext_ = 0 in the H_e_-H_i_-space, which reflect the borders of the response behavior regions in the dynamic function map for the two-population model with disinhibition (Fig 7), were examined and are depicted in Fig 8. It shows that for increasing inhibitory synaptic gain the upper and lower fold bifurcations, which delimit the multistabilty range of the fixed point curve, resemble the three-population model more than the two-population model without disinhibition. Moreover, the upper fold bifurcation remains a saddle node bifurcation for increasing inhibitory synaptic gains and preserves stability of the upper part of the fixed point curve. This is in contrast to both alternative models, for which the saddle-node bifurcation splits into a saddle-saddle and a subcritical Hopf bifurcation (Fig 8). The preserved bistability and the absence of a separatrix (filtering out brief stimuli, see Fig 6) explain the considerably larger range of working memory in the two-population model with disinhibition. In terms of signal flow gating, the two-population model with disinhibition filters stimuli according to their intensity but disregards their temporal consistency and transiency.

**Fig 9:**
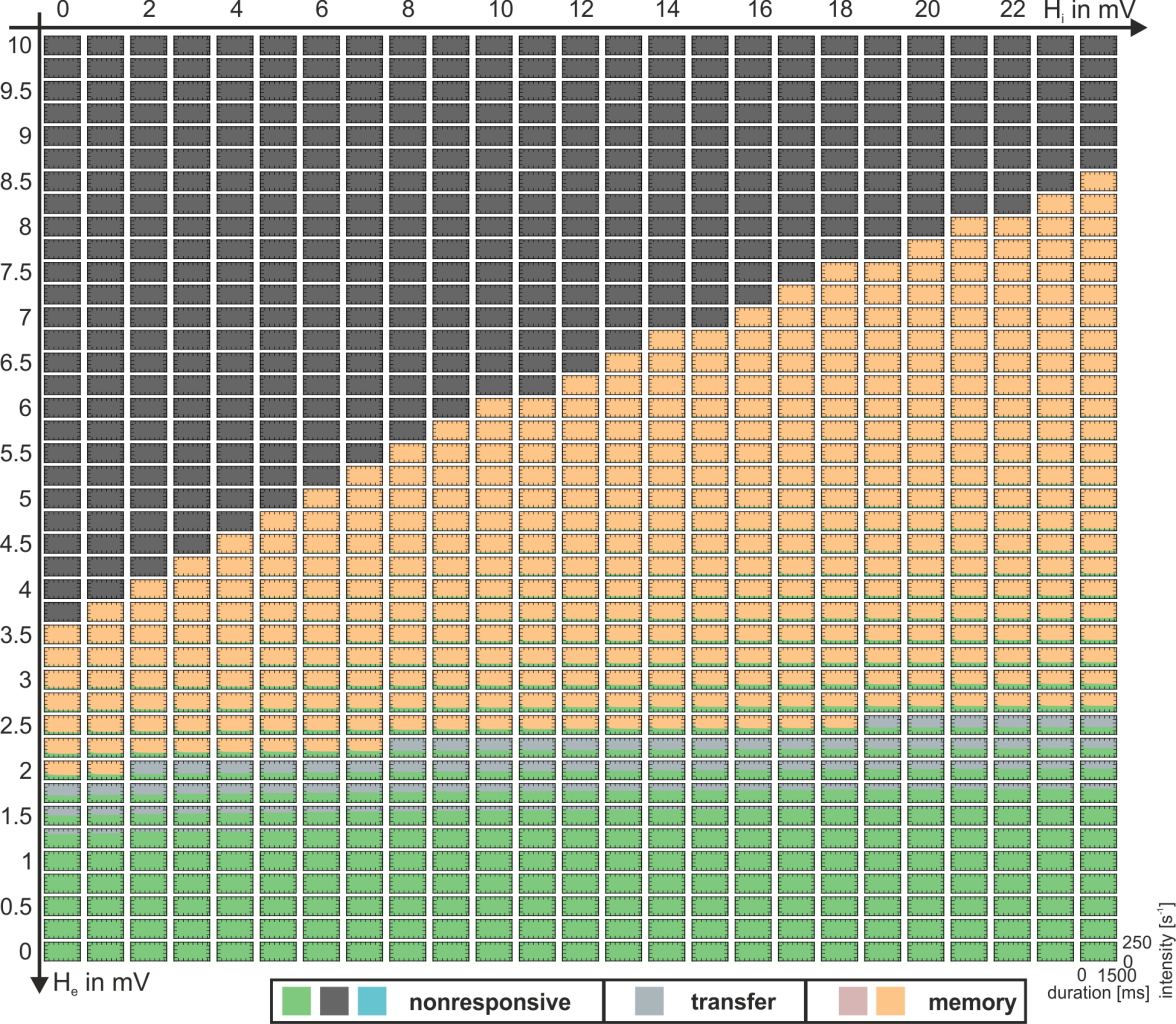
Dynamic function map for the two-population model with disinhibition (see Fig 1D). Collection of characteristic fingerprints for varying excitatory (H_e_) and inhibitory (H_i_) synaptic gains. Color-coded are the observed response behaviors: nonresponsive (bright green and anthracite), transfer (grey), and memory (orange). The variety of observed behaviors is reduced compared to the three-population case (Fig 5). However, all three main types are observable. See S6 Fig for a duplication of this figure, extended by explanatory state space diagrams.

**Fig 10:**
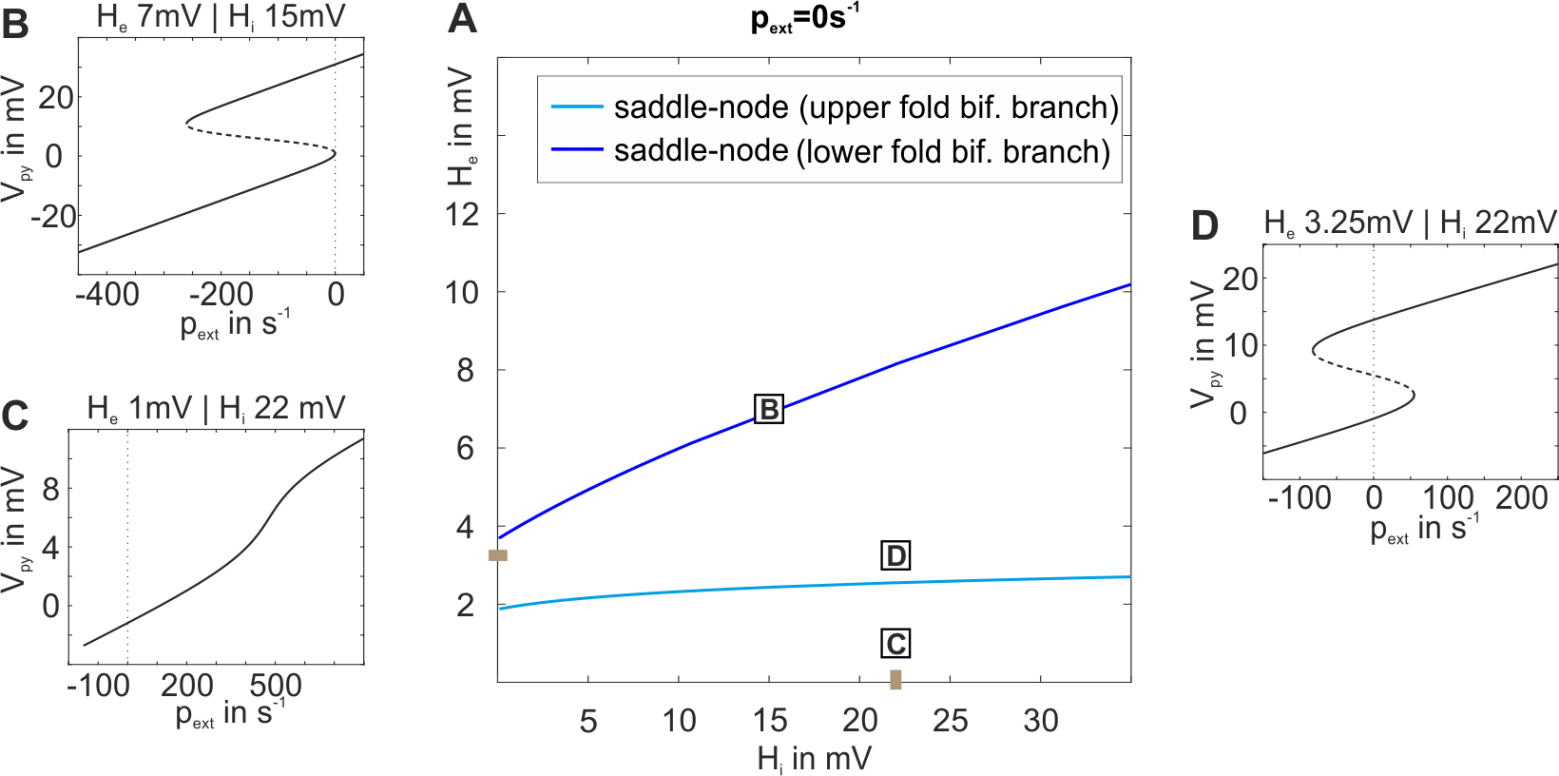
Two parameter bifurcation plot for the two-population model with disinhibition (see Fig 1D). A) The plot characterizes the existing bifurcations at p_ext_ = 0 for the direct excitatory feedback architecture with disinhibition and tracks them through the parameter space spanned by the excitatory and inhibitory synaptic gains H_e_ and H_i_. The region between the upper (cyan line) and lower (blue line) fold bifurcation limits the parameter range for a bistable fixed point curve. These bifurcation branches ranges reflect the borders of nonresponsive, transfer, and memory behavior in Fig 7). B)-D) The single parameter bifurcation plots show the fixed point curve (V_Py_) and local bifurcations along p_ext_ for distinct values of the local network balance.

## Application to sentence processing

We employed the canonical microcircuit to model the cognitive function of sentence processing. During sentence perception, a continuous stream of words is incrementally transformed into a hierarchically organized neural representation reflecting the meaning of the perceived sentence. A reproduction of the sentence after some time necessitates a local representation which stores the words and their relation to each other. In this part of the study we show, how a network composed of canonical microcircuits is able to parse a sentence based on its syntactic information. The selective activation of word-representing neural areas and the defined retention of local information rest upon the basic operations examined above. We further show how an alteration of the network balance perturbs the structure-building process of sentence perception.

In the following we address the question how the ambiguous word information in the exemplary sentence ‘*I hit the thief with the club*’ can be represented in a distributed neural network of canonical microcircuits. This sentence is ambiguous in its syntax: the phrase ‘*with the club*’ can be interpreted as adverbial phrase, that is, further specifying ‘*hit’*, or as adjective phrase, further specifying ‘*the thief’*. We assume that this ambiguity is solved by available contextual information, i.e. prosodic information or specific knowledge concerning the discourse or the topic. In the proposed network model, depicted in Fig 9, each word is represented by a single neural area (i.e., place coding see [49]), modeled through a single canonical microcircuit. These word-representing microcircuits are categorized into modules according to their syntactical role, i.e. subjects, verbs, objects, and their modifiers. The values of the extrinsic inter and intra module connections were optimized by hand to give sensible responses. These values can be found in Fig 10. Further, we assume that the temporal order of the words provides information about the assignment of subject and object [49]. The proposed structure-building computation is based on an input-driven sequential activation of the canonical microcircuits. An activated microcircuit, belonging to a certain word module, transmits its increased firing rate to those modules, which are likely to follow, and differentially pre-activates the respective words by means of weighted connections, creating expectations. In the model, this graded pre-activation corresponds to a shift in the baseline activation of a microcircuit and brings the system closer to the respective fold bifurcation (see section *Principal Dynamics* and Fig 4). In that sense, an activation of the word ‘*eat*’ in the verb module pre-activates the words in the module of verb-modifiers and in the module of objects, but does not activate the words in the module of verbs again (see Fig 9). Subsequent afferent word information can then fully activate the respective microcircuit and continue the structure-building process. The verb and object modifying modules (see Fig 9) allow for competing interpretations of a sentence. Their mutual inhibition in combination with the present level of contextual information ensures that one particular interpretation is supported at a time. The network topology incorporates findings about the phrasal structure of sentences, parsing principles (i.e. *late closure* [50]) and reflects the predictive character of sentence processing.

**Fig 11:**
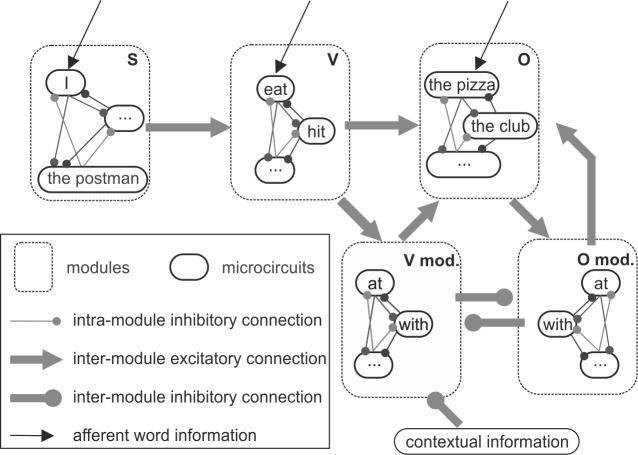
Sentence processing network for sentence comprehension. Afferent word information selectively excites a word-representing canonical microcircuit when the respective word is recognized in primary auditory areas. The activated microcircuit, for example representing the word ‘*I*’ and belonging to the subject module (S), pre-activates words in the connected verb-module (V) and, together with the selective afferent word information, activates another microcircuit (‘*eat*’). Now, words both in the module of verb-modifiers (V mod.) and in the module of objects (O) are differentially pre-activated by weighted connections. Contextual information is proposed to guide this input-driven structure-building process by modulating the excitability of a targeted microcircuit, such as, in our case, through inhibition.

**Fig 12:**
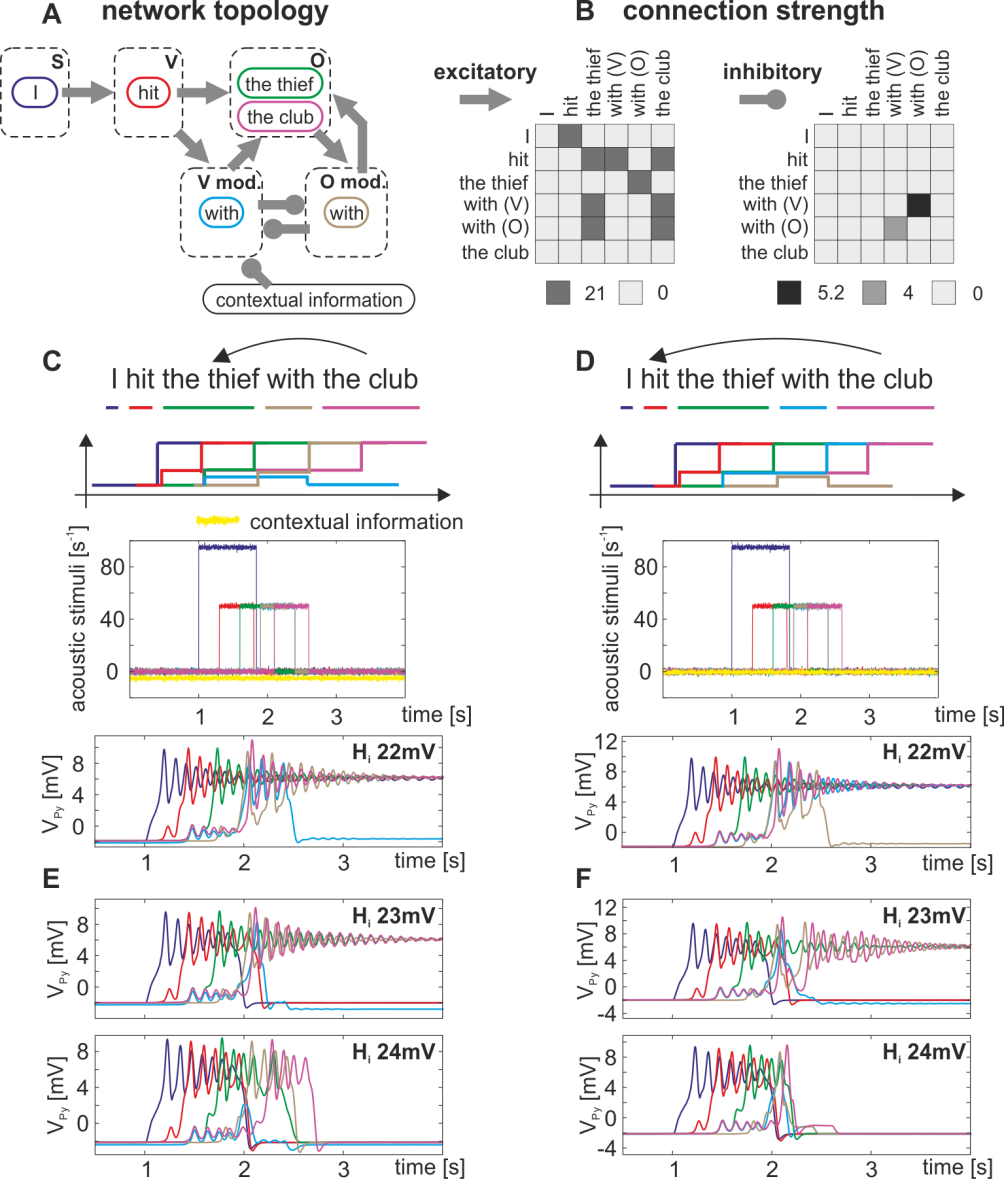
Neural representation of a perceived sentence by a distributed network of six interacting canonical microcircuits. A) The network topology features modules (dashed rectangles) containing the six relevant word-representing canonical microcircuits (solid colored rectangles), which are interconnected through excitatory and inhibitory connections of individual strength (B). The line colors consistently reflect the respective words in all panels. C) The interpretation for which the phrase ‘*with the club*’ refers to an adjective phrase, is guided by contextual information (e.g. knowing that there is a thief bearing a club) which inhibits the module of verb-modifiers. D) For the second interpretation, interpreting ‘*with the club*’ as an adverbial phrase, contextual information is low and the verb-modifying module remains activated. E, F) In case the local network balance of the microcircuits is biased in favor of inhibitory influences, an accurate structure building fails for both interpretations (E & F) and will lead to misinterpretations, i.e. defective word activation traces (top plots), or memory loss, i.e. no lasting activation trace at all (bottom plots).

For the representation of the exemplary sentence ‘*I hit the thief with the club*’, we focused on the relevant words and neglected connections to uninvolved microcircuits. Hence, we set up a network of six interacting canonical microcircuits (see Fig 10A), each modeled by a three-population model (Fig 1B), and their respective connections, see Fig 10B. For the parameters, see Table 1. Ambiguity, i.e. whether the phrase ‘*with the club*’ serves as adjective or adverbial phrase, is resolved by separating verb-modifying from object-modifying modules. Both modules mutually inhibit each other through asymmetrical connections, see Fig 10B. Further, the present level of contextual information guides the structure-building process. In the simulations, contextual information was modeled as an inhibitory noisy signal with a constant offset, which degrades the afferent input, represented by p_ext_ (see Fig 1), of a target area. Two interpretations for the same afferent word information were observable. For the first interpretation (see Fig 10C) word information activates ‘*I*’ and subsequently ‘*hit*’. This pre-activates both the object module and the verb-modifier module. Afferent word information now activates ‘*the thief*’, which pre-activates the object-modifier module. Afferent word information then activates ‘*with*’ in both the verb-modifying and the object-modifying modules, resulting in a competitive interaction, which resolves the ambiguity. During the competition, contextual information inhibits the verb-modifying module. Further afferent word information activates ‘*the club’* and completes the structure-building process. For the second interpretation (see Fig 10D), no specific contextual information is present so that the interpretation proceeds according to the listener’s experience, i.e. the individual ratio of mutual inhibition between the modifiers. Consequently, the verb-modifying inhibits the object-modifying module and ‘*with the club*’ is interpreted as an adverbial phrase.

Several neurological disorders are associated with a disturbed network balance on the level of interacting neural populations [26, 29, 32]. Further, drugs, anesthetics, and other chemicals are known to alter number or efficacy of available neurotransmitter receptors. Valenzuela [51] reports on a perturbation of the network balance in favor of inhibitory influences following alcohol consumption. In the three-population model we replicated this scenario by increasing the inhibitory synaptic gain slightly from H_i_ = 22mV to H_i_ = 23mV (H_i_ = 24mV) and observed a defective sentence representation (see Fig 10E and 10F). Although the representing canonical microcircuits receive the respective afferent word information, they are not able to show the necessary sustained activity.

In summary, a network of interacting canonical microcircuits is able to transform a stream of afferent word information into a representative neural activity trace by means of an input-driven sequential activation. However, if the canonical microcircuits are not able to perform the necessary basic operations, for example due to a detuning of the network balance, this structure building process will fail.

## Discussion

In this study we demonstrate how the local architecture of the cortex, as represented by a canonical microcircuit, implements the basic information processing operations of signal flow gating and working memory. These basic operations form major prerequisites for higher-order cortical computations, such as structure building. We investigated to what extent local topological choices constrain these basic operations. We demonstrated that only models with separate excitatory input and output populations (i.e., the three population model, featuring indirect excitatory feedback) feature all relevant response behaviors simultaneously for a single parameterization. Importantly, the local network balance was shown to be a critical factor for the accessibility of the basic operations and thereby the functional individualization of the microcircuit. We exemplified how a network comprising multiple individualized canonical microcircuits can realize a complex cognitive operation, namely syntax parsing in sentence processing.

In the following we will discuss how our choice of basic operations and the canonical microcircuit model can be considered as a common denominator for various other propositions found in literature. We will argue that neural mass models are a suitable choice for the representation of a canonical microcircuit and point out how our investigations go beyond previous studies on neural mass models. Moreover, we will elucidate the strengths and limitations of the provided example in light of neurolinguistics as well as other computational models of sentence processing.

### Basic operations in canonical microcircuits

The notion of diversified cognitive functions being grounded on a relatively uniform local architecture of the underlying neural substrate supports the idea of canonical microcircuits [6, 52], whose efficient interaction constitutes the processing power of the cerebral cortex [1–3]. So far, neurobiological studies agree upon cortex-wide characteristics such as lamination, biophysical properties of dominant types of neurons, as well as target and source layers of transmitted signals [6, 53]. Note, however, that this concept is not entirely undisputed [54, 55].

Canonical microcircuits have been previously associated with basic operations, or stereotypic functions [9], such as gain control and signal restoration [56], linear (e.g. summation, division, and sign inversion) and nonlinear operations (e.g., winner-takes-all, invariance, and multistability) [7], amplification and signal normalization [55, 57], as well as selectivity and computation of gain [2]. Large-scale spiking neuron networks, explicitly emulating the laminar architecture of a cortical column, have been extensively used to examine the link between stereotypic structure and basic operations in computational models of canonical microcircuits [8, 58–60]. The presence of such operations has also been investigated in mean field models [61–63], which form the basis of the majority of attempts to model neurocognitive experiments (e.g., DCM; [37, 64]). In the present study, we advance the understanding of canonical operations by providing evidence for fundamental basic operations even in a very simple feedback model of a canonical microcircuit. Due to their fundamental character, the identified basic operations (signal flow gating and working memory) imply other formerly described canonical operations (see above), which are more complex and were found in the very specific architectures of a cortical column. It has been established that mean field models can reliably describe the collective behavior of large numbers of neurons [65]. Importantly, the spatial abstractness of the type of model we chose, flexibly reflecting the interaction of a few neurons, neural populations, or entire cortical areas, allows the interpretation of this basic functionality to be relevant at various levels of neural organization, potentially exceeding the level of the cortical column.

Nevertheless, the mesoscopic spatial scale of neural populations, at which the uniformity of canonical microcircuits is arguably established, is well captured by the concept of neural mass models [20, 65]. Here we examine its dynamics in response to a transient input, in the light of variations of the underlying state space, which is most relevant for stimulus-driven information processing.

Our focus on a single microcircuit complements recent network-based developments that also appeal to the notion of modeling canonical microcircuits by neural mass models. It has been studied how the apparent distinction of inter-circuit connections into forward and backward connections, with laminar-specific origins and targets [22, 38, 66], motivates the arrangement of canonical microcircuits in hierarchical models [37, 67, 68]. What has not been studied in these models yet are basic operations at the level of the microcircuit and their sensitivity to topological features and levels of excitation and inhibition. In this study, we investigated stimulation-induced response behaviors in three representative local topologies: (i) a three-population model (Fig 1B), (ii) a two-population model (Fig 1C), and (iii) a two-population model with recurrent inhibitory feedback (Fig 1D). Among the examined feedback architectures and respective parameter ranges we found that only the three-population model (with input to the excitatory interneurons, thus with separate input and output populations) exhibits the coexistence of all three response behaviors for a fixed value of the network balance. That is, only a three-population architecture is capable of selectively blocking, transmitting, or memorizing a stimulus based on its properties. Furthermore, the transfer behavior of the two-population models only depends on the strength of the applied stimuli and not on their duration, as opposed to the three-population model, where it depends on both. In that sense, our results demonstrate that the consideration of an indirect excitatory feedback loop increases the diversity and biological realism of the model’s dynamics in very important ways. As more complex model architectures generally tend to exhibit richer dynamical repertoires, we expect these behaviors also to occur in more detailed models of canonical microcircuits. In particular, our model neglects a direct interaction between the excitatory and inhibitory interneurons, as well as the self-feedback among the excitatory interneurons. Both these connections certainly exist [58]. These features are among the important extensions of the model that need to be investigated in future studies.

In our model of a canonical microcircuit, we described local memory behavior based on bistability. The recurrent and self-sustaining activity is modulated by the network balance and is initiated and terminated by afferent inputs, a behavior that has been shown *in vitro* [35]. In contrast to long-term memory that rests on synaptic plasticity, short-term (or working) memory is thought to rely on mechanisms that do not change the underlying connectivity structure. Alternatively, it has been proposed that working memory may be based on delays and time constants [12, 69], such as *synfire chains* [70], recurrent excitatory networks [71], or cellular properties [72]. In these mechanisms the period of storage (forgetting time) depends on relatively fixed structural aspects of the network, whereas with bistability the item can be kept in memory in principle for any period, until it is actively switched off (e.g., by an impulse to the inhibitory population, see S2 Fig).

Neural functions should be robust to noise but sensitive to afferent input at the same time. Signals can be distinguished from noise by their higher amplitude and temporal smoothness. In our model, with respect to amplitude, the distance between the working point and the fold bifurcation should be larger than the noise and smaller than the signal. We demonstrated that this distance is effectively controlled by the local network balance, which is therefore a key parameter for governing the tradeoff between robustness to noise and sensitivity to stimuli (e.g., S4 Fig). With respect to temporal smoothness, only the three-population model offers a selection mechanism. We established that the model keeps a stimulus in memory only if it lasts long enough, that is, it exhibits a large degree of temporal smoothness.

In addition to the concept of functional diversification by topology [3], we showed that a variation of the local network balance is also a biologically plausible means to individualize local functionality. This local tuning determines whether a neural area will selectively forward an impulse (transfer behavior), switch into a higher persistent activation (memory behavior), or not respond to the impulse at all (non-responsive behavior). We note that in this case we have treated the local network balance as a lumped parameter that actually encapsulates a large number of physiological and anatomical properties and mechanisms, such as neurotransmitter kinetics and neuroreceptor densities, dendritic arborization, synaptic and extra-cellular ionic dynamics and local and non-local network connectivities, to name but a few [73, 74]. Because of this ambiguity, and as discussed previously, the concept of network balance is very difficult to quantify. In this research, we have used a simple definition in order to demonstrate as a proof of concept that the local network balance does provide a fundamental means of individualizing local microcircuit functionality.

### Application of the canonical microcircuit to sentence processing

The notion of an extensive network of similar microcircuits supporting cortical function has been put forward in specialized neurocognitive theories. For example, several proposals have been put forward to provide mechanistic insight into the processing of language [1, 49, 61, 62, 75]. It has also been associated with organizing principles such as place coding, where a neural area represents an abstract processing element [49]. In this study, we employed canonical microcircuits to represent constituents of perceived sentences, reflecting the principle of place coding. Single word-representing canonical microcircuits were grouped into modules according to their syntactic role (e.g., subject, object) rather than to their word category (e.g., noun, pronoun). We describe a computational mechanism of input-driven functional binding of discrete elements. This enables the generation of infinite sequences out of a limited number of discrete elements, a concept referred to as *infinite recursion* [3]. The proposed generative structure building mechanism might be called *dynamic recruitment* to emphasize the freedom of the structure concerning its number and order of elements.

The proposed computational mechanism is applied to syntax-parsing, that is, the grouping of a continuous stream of words into a hierarchical structure of sentence constituents. We constrain the structure-building mechanism by fixing the underlying wiring of interacting neural areas. The characteristics of the network, that is, its topology and connection weights, reflect the rules of syntax, which are likely to be established during language learning. The basic operations provided by the canonical microcircuit are crucial for this structure-building mechanism. Clearly, an efficient short-term memory mechanism is needed to implement temporal integration of real-time sequences. The working memory mechanism of our canonical microcircuit provides fast encoding and flexible holding times. The latter is especially relevant in order to account for varying speeds of speech and different sentence lengths. The signal flow gating ensures that words are activated only if they were both pre-activated (expected) by the sentence structure and recognized from the input stream. As these basic operations have been shown to crucially depend on the network balance, altering this parameter leads to failure of the global network operation. In the current implementation of the language network the coexistence of memory and transfer behavior, which is the hallmark of the three population model, is not a necessary ingredient. Thus, also a two population model could have been used. However, for further elaboration of the model, the specific properties of the three population model might become relevant. For example, due to unspecific afferent word information single words can, in principle, occur multiple times (e.g. the word drink as subject, verb, object). To prevent a simultaneous sustained activation of multiple canonical microcircuits (which would saturate the nodes and make them unavailable for later activation), the local circuit needs to feature transfer behaviors provided by the three population model.

During the process of sentence comprehension the initial syntax parsing, addressed in this study, is proposed to be followed by a thematic role assignment and a collective assessment of syntactic and semantic information [76]. Besides pragmatic information, prosodic information is also proposed to guide sentence comprehension [77]. In our model we combine all types of such additional information into the more general notion of contextual information.

The proposed network model reflects characteristics of a *serial*, *syntax-first model* by constructing the simplest syntactic structure on the basis of word-category information, independent of lexical-semantic information [76]. The model also reflects characteristics of the *constraint-satisfaction models* [78] by incorporating nonstructural factors into the network topology. Such factors include the frequency of a particular structure or its semantic plausibility. In further agreement with the concept of constraint-satisfaction models, the proposed network model is able to account for syntactic ambiguities (see Fig 10). We also assume that perception and identification of word information informs, but is conceptually distinct from, the structure-building computations. The incoming auditory information is recognized as a word [79], which is then inputted into neural areas that are recruited during syntax parsing.

In extension to a similar model [49], our model considers modifiers of verbs and objects. However, in the mechanism proposed here, structure-building still strongly rests on temporal word orders, i.e. subject-verb-object. In addition to word category information, a successful syntax parsing would also need to account for morphosyntactic features, such as gender, prefixes or cases. How these features are incorporated is an open question and would require further study.

One aim of our study was to pave the way for a mechanistic understanding of sentence processing in compliance with neuropsychological theories. This is opposed to the field of computational linguistics, which aims to optimize spoken human-machine interaction while sacrificing biological plausibility. Further, we would like to emphasize the developmental character of our model and are aware of several short-comings, some of which we address here:

#### At the word level

Multiple mentions of the same word within a sentence are difficult to deal with. As soon as a word within a distinct module is activated it needs to be deactivated before it can be activated again. Although a deactivation mechanism is implicitly included in our model, through a brief impulse to the IIN, an online deactivation of a single word would interrupt the representative activation trace. Further, the summarizing effect of multiple pre-activations will eventually lead to an activation of an area, even if the word is not present at the input. An extension of the model could scale the pre-activation level relative to the activation threshold, so that a word is ‘on the tip of one’s tongue’ but not activated yet.

#### At the sentence level

Again, representations need to be deactivated, before a following sentence could be represented. To follow a conversation it is necessary to activate a sequence of words and their associated word-webs [80]. These webs decay slowly so that information spanning multiple sentences can be linked together through associative processes. Finally, it has been shown previously that networks with too many activated nodes tend to become unstable and thereby destroy information stored in the network state [42], which could be relevant for long or complex sentences.

## Conclusion

Our results support the concept of computational primitives [7] or stereotypic functions [9] and identify a minimum model structure for a canonical microcircuit that supports these functions. They further corroborate, at the local level, the crucial role of the network balance for the information processing capacity of neural networks. We conclude that our findings lend support to the connectionist idea that higher brain function arises from networks of relatively similar, though individually tunable, canonical microcircuits.

## Supporting information

S1 Fig Increasing recurrent inhibitory self-feedback in the two-population model. A) The two parameter bifurcation plot tracks the occurring bifurcations along p_ext_, when recurrent inhibitory self-feedback N_II_ is increased (i.e., b_2_ is set to zero, see Fig 1D). The network balance was held constant at values H_e_ = 3.25mV and H_i_ = 22mV. B-E) The single parameter bifurcation plot show the fixed point curve (V_Py_) and local bifurcations along p_ext_ for different values of N_II_. F) Fixed point curves for the firing rate of the Py, φ(t), along p_ext_ for different values of the local network balance.
(TIF)

S2 Fig Deactivation diagram for brief input to the IIN. Sufficiently long and strong impulses to the IIN (blue area) are able to deactivate the system, i.e. transfer the system from the active to the inactive state.
(TIF)

S3 Fig Phase-dependency between the system’s intrinsic oscillation and stimulus switch off time. The diagram shows a collection of system responses to stimuli of constant intensity (100s^-1^) and stimulus durations ranging between 600-750ms, where the stimulus offset times are marked by vertical lines. Blue lines denote stimuli responses, for which the system will eventually return to the inactive state after the stimulus was switched off (i.e. transfer behavior). Red lines denote stimuli and responses for which the system was able to remain activated (i.e. memory behavior). Whether the system remains in the activated state depends on the time point of stimulus-offset relative to the phase of the oscillatory response. This behavior arises from the distinct trajectory of the system in the state space when the stimulus is on. As soon as the stimulus is switched off, the system’s phase point is either within the basin of attraction of the stable focus of the upper branch of the fixed point curve or will be attracted to the stable node of the lower branch of the fixed point curve (compare to Fig 4). Both basins of attraction are separated by the irregularly shaped separatrix arising from the unstable Hopf bifurcation (see projection in Fig 4A). The longer the stimulus duration, the more time does the system have in order to settle down to the fixed point curve, which increases the likeliness of residing in the basin of attraction of the upper branch fixed point (the memory behavior) and causes the wider stripes for larger stimulus durations in Fig 3B.
(TIF)

S4 Fig Dynamic function map for the indirect excitatory feedback architecture (see Fig 1B). A) Collection of characteristic fingerprints for varying excitatory (H_e_) and inhibitory (H_i_) synaptic gains. Colors code the observed response behaviors: nonresponsive (bright green, anthracite and cyan regions), transfer (grey regions), and memory (orange and rose regions). The local network balance controls the dominance of the behaviors and tunes the criticality of the system. B-J) Exemplary parameterizations featuring fingerprints, time courses, and projections thereof in a bifurcation plot.
(TIF)

S5 Fig Dynamic function map for the direct excitatory feedback architecture (see Fig 1C). A) Collection of characteristic fingerprints for varying excitatory (H_e_) and inhibitory (H_i_) synaptic gains. Colors code the observed response behaviors: nonresponsive (bright green and anthracite), transfer (grey), and memory (orange). The variety of observed behaviors is reduced compared to the three-population case (S4 Fig). However, all three main types are observable. B)-G) Selected parameterizations featuring fingerprints, time courses, and projections thereof in a bifurcation plot.
(TIF)

S6 Fig Dynamic function map for the two-population model with disinhibition (see Fig 1D). A) Collection of characteristic fingerprints for varying excitatory (H_e_) and inhibitory (H_i_) synaptic gains. Color-coded are the observed response behaviors: nonresponsive (bright green and anthracite), transfer (grey), and memory (orange). The variety of observed behaviors is reduced compared to the three-population case (Fig 5). However, all three main types are observable. B)-E) Selected parameterizations featuring fingerprints, time courses, and their projections in a bifurcation plot.
(TIF)

S1 File Gradual mapping of two excitatory populations into a single one by introducing self-feedback N_PP_. (PDF)
