# Supplementary figures and images for: A model of individualized canonical microcircuits supporting cognitive operations
Source: PLoS One. 2017 Dec 4;12(12):e0188003. doi: 10.1371/journal.pone.0188003 (PMC5714354; doi:10.1371/journal.pone.0188003)

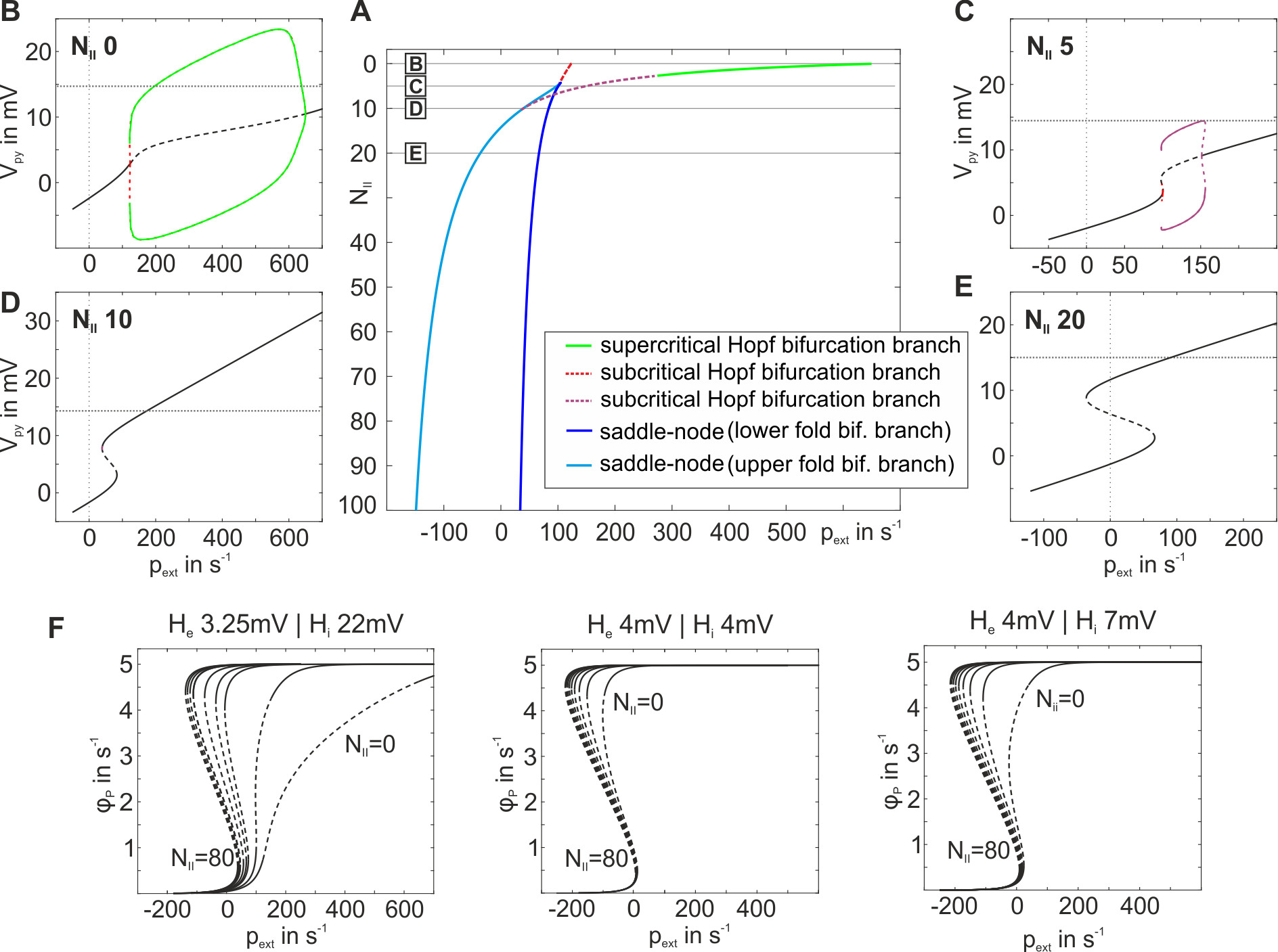

Supplement: S1 Fig — A) The two parameter bifurcation plot tracks the occurring bifurcations along pext, when recurrent inhibitory self-feedback NII is increased (i.e., b2 is set to zero, see Fig 1D). The network balance was held constant at values He = 3.25mV and Hi = 22mV. B-E) The single parameter bifurcation plot show the fixed point curve (VPy) and local bifurcations along pext for different values of NII. F) Fixed point curves for the firing rate of the Py, φ(t), along pext for different values of the local network balance. (TIF) [file pone.0188003.s001.tif]

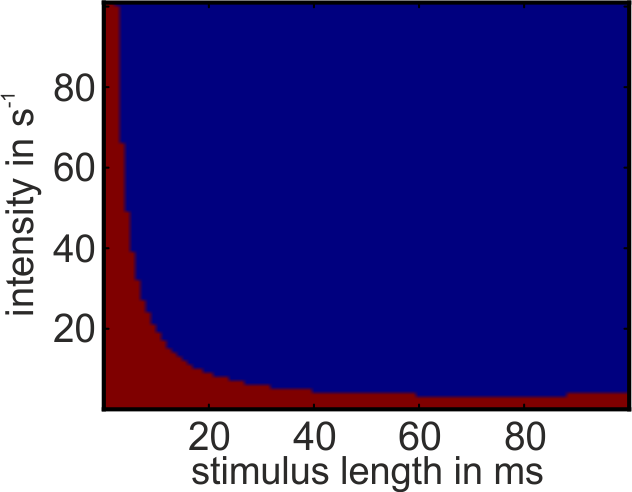

Supplement: S2 Fig — Sufficiently long and strong impulses to the IIN (blue area) are able to deactivate the system, i.e. transfer the system from the active to the inactive state. (TIF) [file pone.0188003.s002.tif]

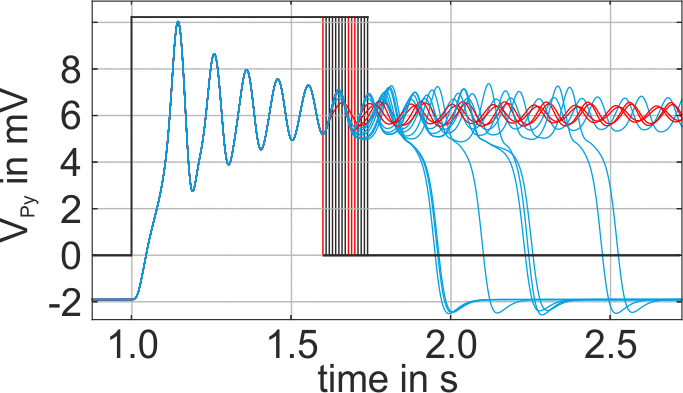

Supplement: S3 Fig — The diagram shows a collection of system responses to stimuli of constant intensity (100s-1) and stimulus durations ranging between 600-750ms, where the stimulus offset times are marked by vertical lines. Blue lines denote stimuli responses, for which the system will eventually return to the inactive state after the stimulus was switched off (i.e. transfer behavior). Red lines denote stimuli and responses for which the system was able to remain activated (i.e. memory behavior). Whether the system remains in the activated state depends on the time point of stimulus-offset relative to the phase of the oscillatory response. This behavior arises from the distinct trajectory of the system in the state space when the stimulus is on. As soon as the stimulus is switched off, the system’s phase point is either within the basin of attraction of the stable focus of the upper branch of the fixed point curve or will be attracted to the stable node of the lower branch of the fixed point curve (compare to Fig 4). Both basins of attraction are separated by the irregularly shaped separatrix arising from the unstable Hopf bifurcation (see projection in Fig 4A). The longer the stimulus duration, the more time does the system have in order to settle down to the fixed point curve, which increases the likeliness of residing in the basin of attraction of the upper branch fixed point (the memory behavior) and causes the wider stripes for larger stimulus durations in Fig 3B. (TIF) [file pone.0188003.s003.tif]

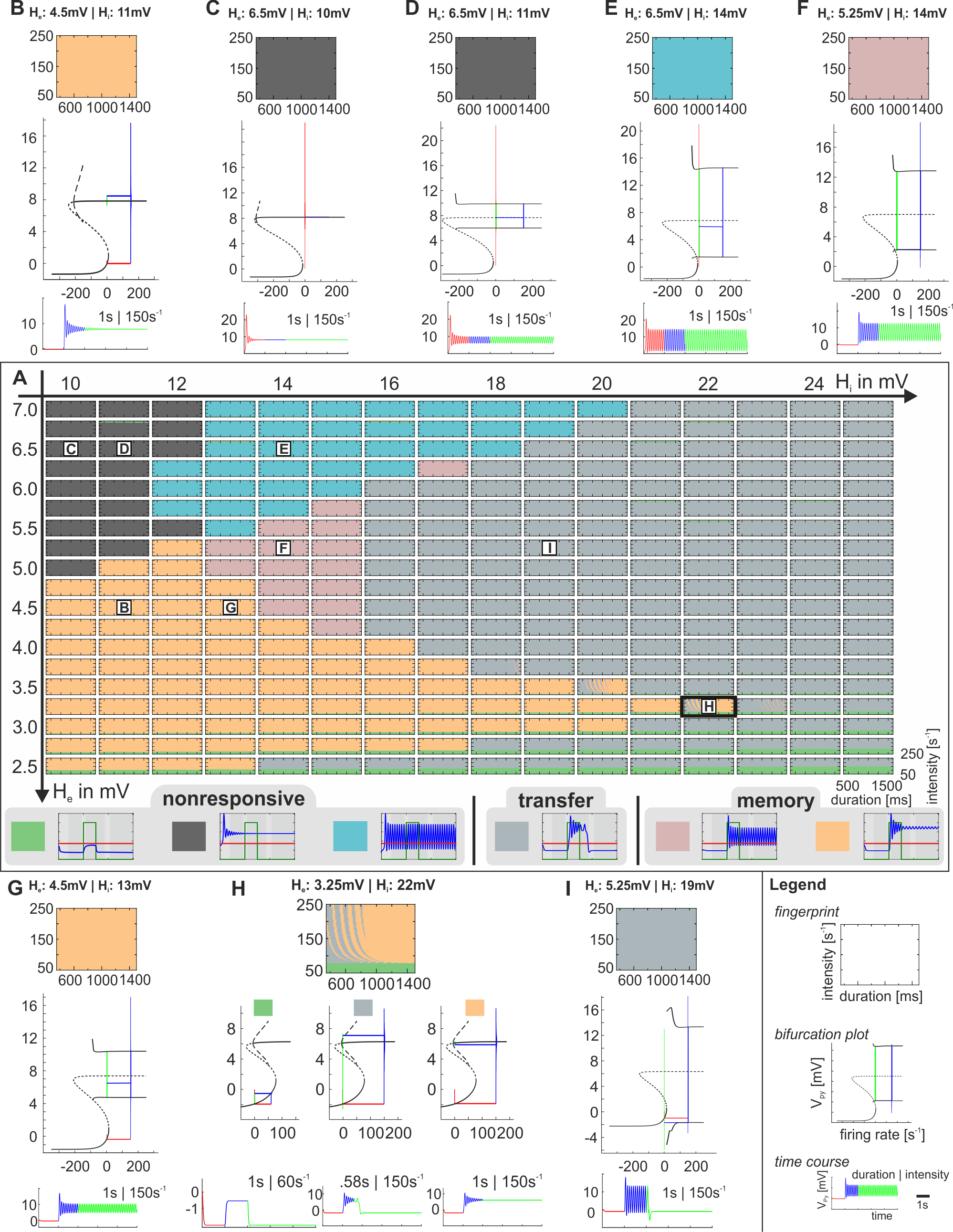

Supplement: S4 Fig — A) Collection of characteristic fingerprints for varying excitatory (He) and inhibitory (Hi) synaptic gains. Colors code the observed response behaviors: nonresponsive (bright green, anthracite and cyan regions), transfer (grey regions), and memory (orange and rose regions). The local network balance controls the dominance of the behaviors and tunes the criticality of the system. B-J) Exemplary parameterizations featuring fingerprints, time courses, and projections thereof in a bifurcation plot. (TIF) [file pone.0188003.s004.tif]

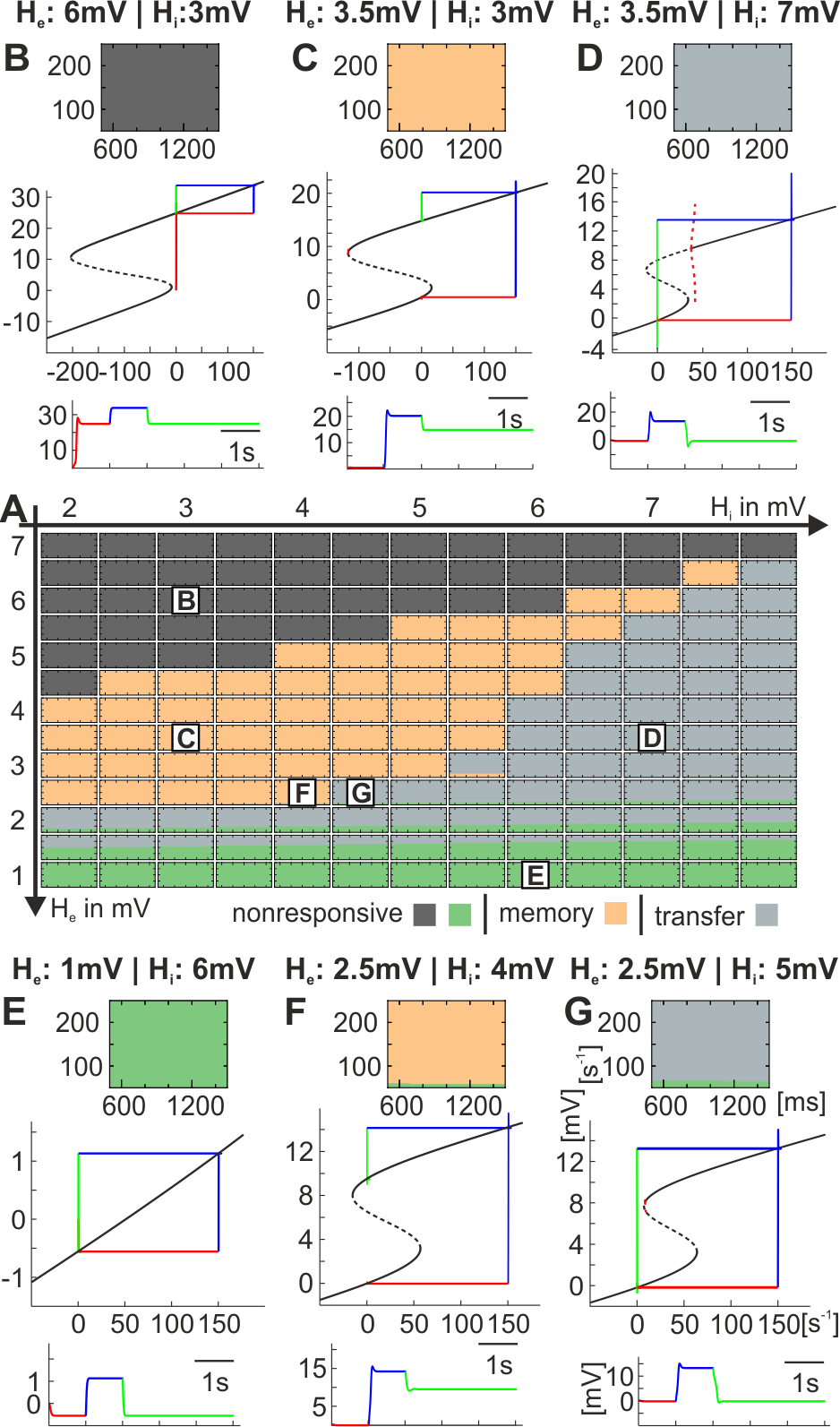

Supplement: S5 Fig — A) Collection of characteristic fingerprints for varying excitatory (He) and inhibitory (Hi) synaptic gains. Colors code the observed response behaviors: nonresponsive (bright green and anthracite), transfer (grey), and memory (orange). The variety of observed behaviors is reduced compared to the three-population case (S4 Fig). However, all three main types are observable. B)-G) Selected parameterizations featuring fingerprints, time courses, and projections thereof in a bifurcation plot. (TIF) [file pone.0188003.s005.tif]

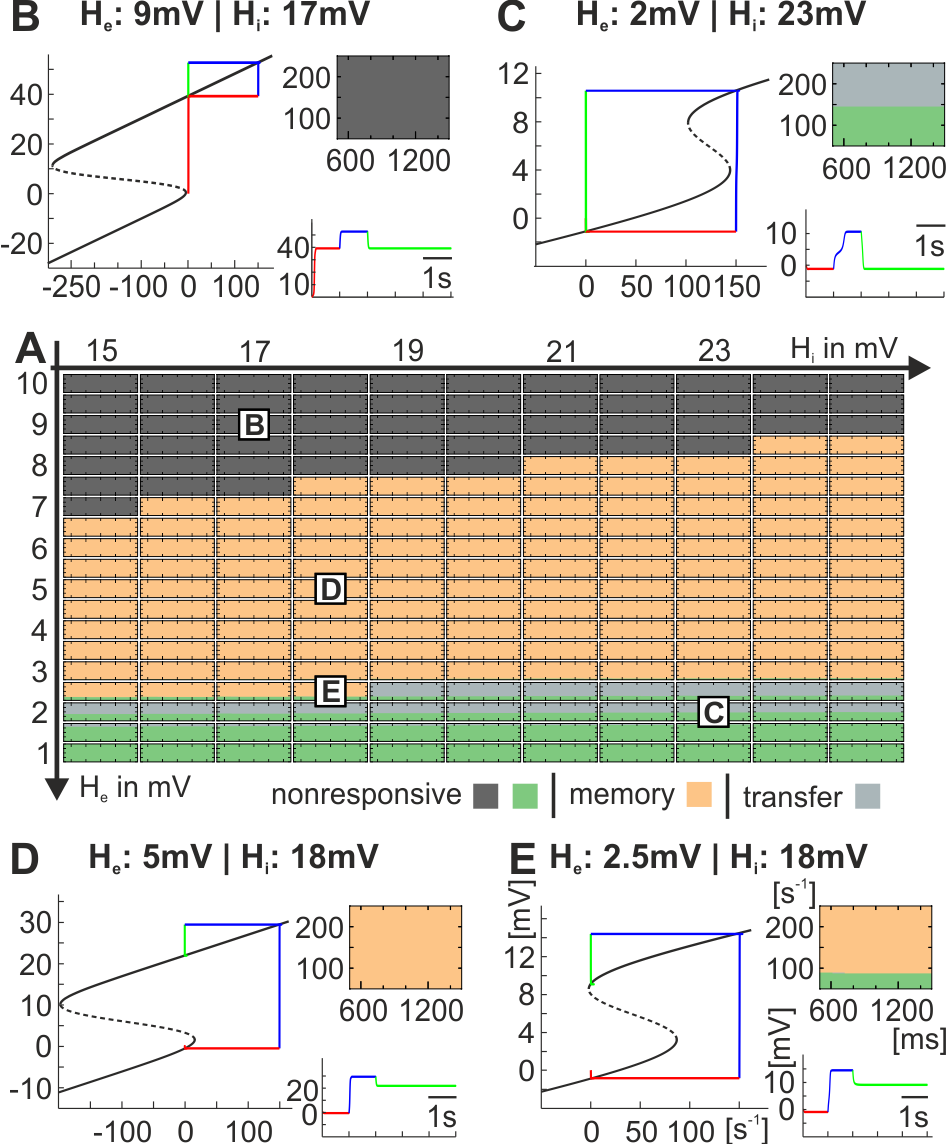

Supplement: S6 Fig — A) Collection of characteristic fingerprints for varying excitatory (He) and inhibitory (Hi) synaptic gains. Color-coded are the observed response behaviors: nonresponsive (bright green and anthracite), transfer (grey), and memory (orange). The variety of observed behaviors is reduced compared to the three-population case (Fig 5). However, all three main types are observable. B)-E) Selected parameterizations featuring fingerprints, time courses, and their projections in a bifurcation plot. (TIF) [file pone.0188003.s006.tif]
