# Supplementary material for: A model of individualized canonical microcircuits supporting cognitive operations
Source: PLoS One. 2017 Dec 4;12(12):e0188003. doi: 10.1371/journal.pone.0188003 (PMC5714354; doi:10.1371/journal.pone.0188003)
Supplement: S1 File — (PDF) [file pone.0188003.s007.pdf]

S1 File. Gradual mapping of two excitatory populations into a single one by introducing self-feedback N_{PP}.

When mapping two excitatory populations into a single excitatory population we demand that the mapping must conserve: i) the total number of neurons in the system, ii) the sum of flowing currents, and iii) all connections between single neurons.

In the two-population case, we assume that the EIN population contains M_E neurons and the Py population contains M_P neurons. **We interpret the connectivity gain N_{ab} as the average number of neurons of population *b*, which a single neuron in population *a* receives input from**. When mapping the two-populations onto a single population we gradually merge more and more neurons, a fraction denoted by $\beta=\alpha\cdot M_E$, from EIN with neurons from Py into a growing population P'. α denotes a linear scaling factor ranging from 0 to 1.

When $\alpha=0$, $\beta=\alpha\cdot M_E=0$, no neurons have been transferred yet and we observe the regular two excitatory populations with default values for N_{EP} and N_{PE} (top panel in Fig 1A)

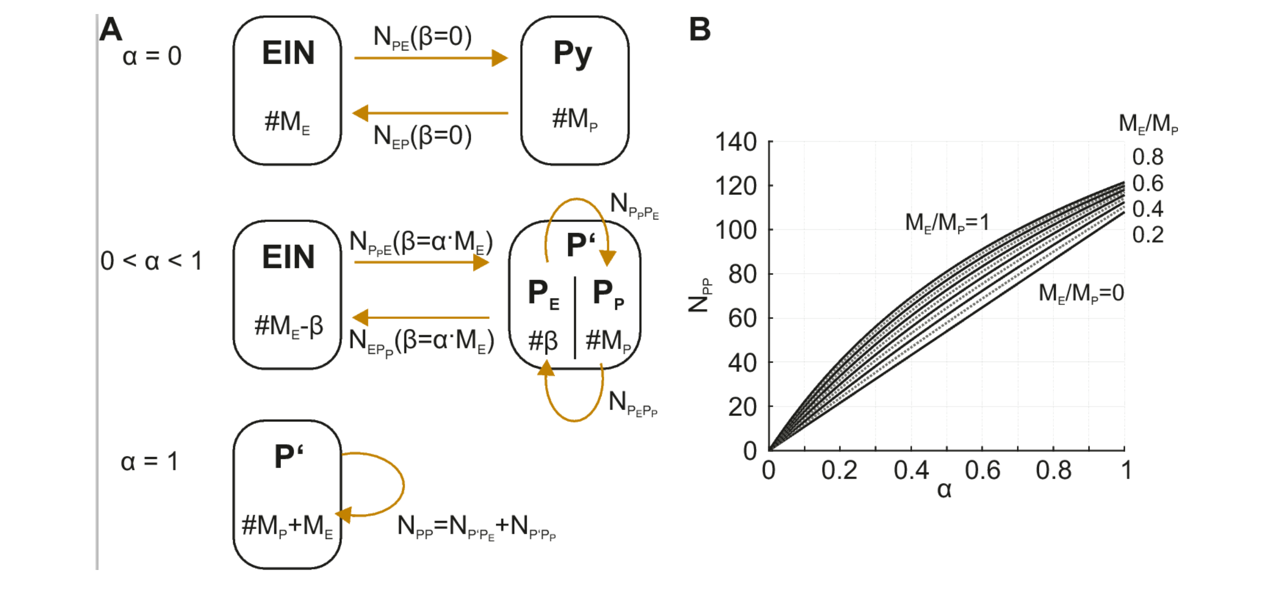

Fig 1. Mapping two excitatory populations onto a single population. A) Top: For α=0, the two excitatory populations EIN, featuring a number of M_E neurons, and Py, featuring a number of M_P neurons, are coupled through connections of strength N_{PE} and N_{EP}, respectively. Middle: For 0<α<1, a fraction of neurons, P_E of number $\beta=\alpha\cdot M_E$, which was previously associated with the EIN, merges with former neurons of the Py, P_P, to a growing population P'. While the connection strength N_{EP_P} stays the same (P_P still fire), internal feedback $N_{P_EP_P}$, is established within population P'. Further, feedback from EIN to P', N_{P_PE}, is reduced in favor of self-connections, $N_{P_PP_E}$, within population P'. Bottom: For α=1, all former EIN neurons are merged with former Py neurons constituting population P', which features self-feedback of strength N_{PP}. B) Development of N_{PP} over α, implying a co-existence of direct and indirect excitatory feedback in the model, for various proportions of excitatory interneurons and pyramidal cells M_E/M_P.

When 0<α<1, neurons of number $\beta=\alpha\cdot M_E$ are transferred from EIN to P', which are denoted P_E. Within P' there is now a fraction P_P of number M_P and a fraction P_E of number β. Thus, the total number of neurons in EIN is M_E-β and the total number of neurons in P' is M_P+ β (middle panel in Fig. 1A). The connectivity has changed in the following way: connections from EIN to P_P

(former N_{PE}) is scaled by a factor $(M_E$-β$)/M_E$, because less neurons in EIN are available to project onto P' (source fraction). This is reflected by:

$$N_{P_pE}(\beta = \alpha \cdot M_E) = \frac{M_E - \beta}{M_E} \cdot N_{PE}(\beta = 0) = (1 - \alpha) \cdot N_{PE}(\beta = 0)\,. \quad (1)$$

Connectivity from P_P to EIN (former N_{EP}) remains the same, because the number of neurons, which originally projected from Py to EIN, now bounded in P_P, remains constant at M_P (source fraction). Thus, N_{EP_P} reads:

$$N_{EP_P}(\beta = \alpha \cdot M_E) = N_{EP}(\beta = 0)\,. \quad (2)$$

The transfer of neurons gives rise to self-connections within P', N_{PP}, which are represented by the sum of projections from P_P to P_E ($N_{P_EP_P}$) and projections from P_E to P_P ($N_{P_PP_E}$). For $N_{P_EP_P}$, like in the fully separated case, P_P (source fraction) still project onto P_E:

$$N_{P_EP_P}(\beta = \alpha \cdot M_E) = N_{EP}(\beta = 0)\,. \quad (3)$$

For $N_{P_PP_E}$ the P_E project onto P_P, but are scaled by a factor β/M_E reflecting their reduced number (source fraction) as in:

$$N_{P_PP_E}(\beta = \alpha \cdot M_E) = \frac{\beta}{M_E} \cdot N_{PE}(\beta = 0)\,. \quad (4)$$

As the population P' is growing, the incoming input is distributed among more and more neurons. This is reflected by an additional scaling of the connectivity gains (distribution factor). Connections from EIN, that is N_{P_PE} (equation 1), are scaled by a distribution factor $M_P/(M_P+\beta)$. Thus $N_{P'E}$ reads:

$$N_{P'E}(\beta=\alpha\cdot M_E)=\frac{M_P}{M_P+\beta}\cdot(1-\alpha)\cdot N_{PE}(\beta=0)=(1-\alpha)\cdot\frac{M_P}{M_P+\alpha\cdot M_E}\cdot N_{PE}(\beta=0)=\cdot\frac{1-\alpha}{1+\alpha\cdot M_E/M_P}\cdot N_{PE}(\beta=0)$$

. (5)

Within P', $N_{P_E P_P}$ (equation 3) is scaled by a distribution factor $\beta/(M_P+\beta)$. Thus, $N_{P'P_P}$ reads:

$$N_{P'P_P}(\beta=\alpha\cdot M_E)=\frac{\beta}{M_P+\beta}\cdot N_{EP}(\beta=0)=\frac{\alpha}{M_P/M_E+\alpha}\cdot N_{EP}(\beta=0)\,. \qquad (6)$$

Also within P', $N_{P_P P_E}$ is scaled by a distribution factor $M_P/(M_P+\beta)$. Thus, $N_{P'P_E}$ reads:

$$N_{P'P_E}(\beta=\alpha\cdot M_E)=\frac{M_P}{M_P+\beta}\cdot\frac{\beta}{M_E}\cdot N_{PE}(\beta=0)=\frac{\alpha}{1+\alpha\cdot M_E/M_P}\cdot N_{PE}(\beta=0)\,. \qquad (7)$$

Thus, the total number of self-connections in P' reads:

$$N_{PP}(\beta=\alpha\cdot M_E)=N_{P'P_E}+N_{P'P_P}=\frac{\alpha}{1+\alpha\cdot M_E/M_P}\cdot N_{PE}(\beta=0)+\frac{\alpha}{M_P/M_E+\alpha}\cdot N_{EP}(\beta=0)\,. \qquad (8)$$

Assuming that the ratio between number of excitatory interneurons and number of pyramidal cells, M_E/M_P, is 0.25 (1), and that N_{PE}=108 and N_{EP}=135, N_{PP} equals 113.4 in case of the completed mapping (see bottom panel in Fig 1A). The diagram in Fig 1B shows the function $N_{PP}(\alpha)$, for different M_E/M_P. Consequent changes in the bifurcation structure of the system are reported in Fig 2.

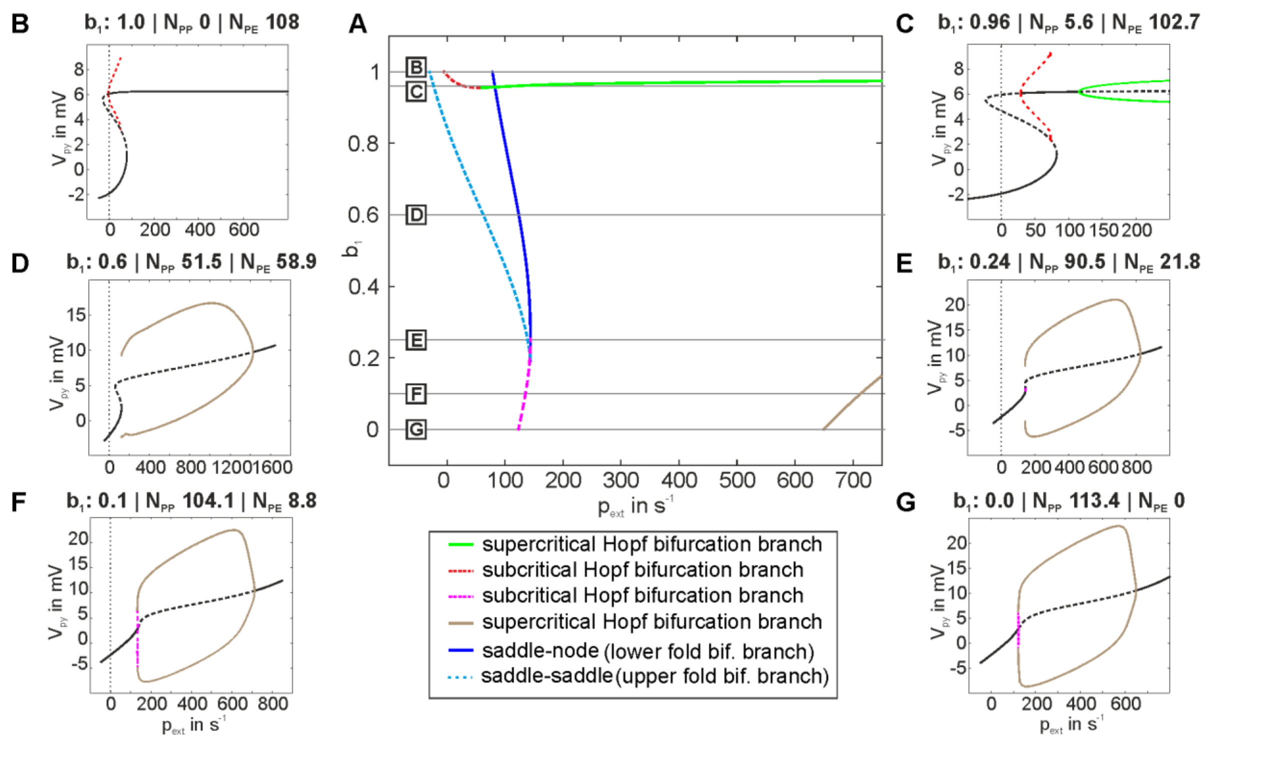

Fig 2. Gradual Transformation of the indirect excitatory feedback to a direct excitatory feedback. A) The two parameter bifurcation plot tracks the occurring bifurcations along p_{ext}, when b_1 is changed from 1 (three-population model) to 0 (two-population model, see main text). For this mapping, the network balance was held constant at values H_e=3.25mV and H_i=22mV. This transformation reflects the mapping described in Fig 1A. B-G) The single parameter bifurcation plots show the fixed point curve (V_{Py}) and local bifurcations along p_{ext} for different values of b_1.

References

1. Meyer HS, Wimmer VC, Oberlaender M, de Kock CP, Sakmann B, Helmstaedter M. Number and laminar distribution of neurons in a thalamocortical projection column of rat vibrissal cortex. Cerebral cortex (New York, NY : 1991). 2010;20(10):2277-86.
